# Two-Terminal Lithium-Mediated Artificial Synapses with Enhanced Weight Modulation for Feasible Hardware Neural Networks

**DOI:** 10.1007/s40820-023-01035-3

**Published:** 2023-03-21

**Authors:** Ji Hyun Baek, Kyung Ju Kwak, Seung Ju Kim, Jaehyun Kim, Jae Young Kim, In Hyuk Im, Sunyoung Lee, Kisuk Kang, Ho Won Jang

**Affiliations:** 1 Department of Materials Science and Engineering, Research Institute of Advanced Materials, Seoul National University, Seoul, 08826 Republic of Korea; 2 Advanced Institute of Convergence Technology, Seoul National University, Suwon, 16229 Korea

**Keywords:** Artificial synapse, Neuromorphic, Li-based, Two-terminal, Synaptic plasticity

## Abstract

Recently, artificial synapses involving an electrochemical reaction of Li-ion have been attributed to have remarkable synaptic properties. Three-terminal synaptic transistors utilizing Li-ion intercalation exhibits reliable synaptic characteristics by exploiting the advantage of non-distributed weight updates owing to stable ion migrations. However, the three-terminal configurations with large and complex structures impede the crossbar array implementation required for hardware neuromorphic systems. Meanwhile, achieving adequate synaptic performances through effective Li-ion intercalation in vertical two-terminal synaptic devices for array integration remains challenging. Here, two-terminal Au/Li_*x*_CoO_2_/Pt artificial synapses are proposed with the potential for practical implementation of hardware neural networks. The Au/Li_*x*_CoO_2_/Pt devices demonstrated extraordinary neuromorphic behaviors based on a progressive dearth of Li in Li_*x*_CoO_2_ films. The intercalation and deintercalation of Li-ion inside the films are precisely controlled over the weight control spike, resulting in improved weight control functionality. Various types of synaptic plasticity were imitated and assessed in terms of key factors such as nonlinearity, symmetricity, and dynamic range. Notably, the Li_*x*_CoO_2_-based neuromorphic system outperformed three-terminal synaptic transistors in simulations of convolutional neural networks and multilayer perceptrons due to the high linearity and low programming error. These impressive performances suggest the vertical two-terminal Au/Li_*x*_CoO_2_/Pt artificial synapses as promising candidates for hardware neural networks

**Supplementary Information:**

The online version contains supplementary material available at 10.1007/s40820-023-01035-3.

## Highlights

- The Li-mediated artificial synapses with a vertical two-terminal configuration capable of various synaptic behaviors, including bio-plausible synaptic plasticity, were successfully demonstrated for the first time and thoroughly explored
- Synaptic characteristics based on the progressive dearth of Li in Li_*x*_Co_2_ films are precisely controlled over the weight control spike, achieving extraordinary weight control functionality.
- In artificial neural network simulation, Li_*x*_CoO_2_-based neuromorphic system showed excellent accuracy comparable to the theoretical maximum due to the low nonlinearity and programming error, suggesting feasibility of hardware neural network implementation.

**Supplementary Information:**

The online version contains supplementary material available at 10.1007/s40820-023-01035-3.

## Graphical abstract

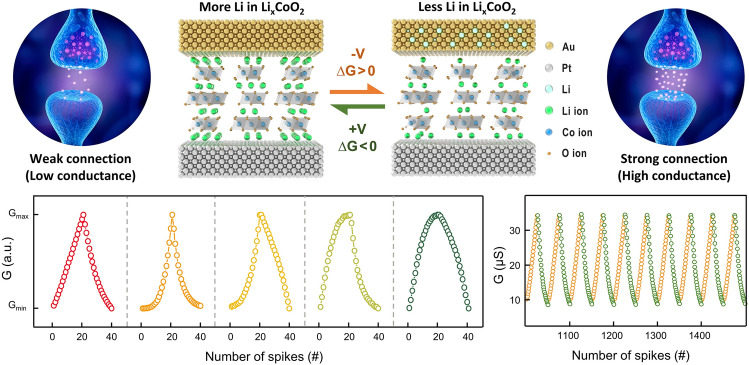

## Introduction

With the advent of the machine learning era, human-generated unstructured data such as text, images, and audio are exploding. Processing vast amounts of data with a conventional computing system based on the von Neumann architecture has reached its limits [1, 2]. The challenges for modern computing systems originate from the reduced efficiency of energy and throughput caused by constant data transfer between memory and processing units, well known as the von Neumann bottleneck [3, 4]. Neuromorphic computing inspired by the functionality of the human brain has received considerable attention as one of the ways to achieve technical requirements to overcome von Neumann bottlenecks [5–8]. Computing technology that executes massively parallel processing in an energy-efficient manner can handle such unstructured data productively. The brain-inspired computing system can be realized as a hardware implementation of a neural network platform made up of combinations of numerous artificial neurons and synapses [9, 10]. In neuroscience, a synapse is a functional junction between two neurons that transmits signals from the pre-synaptic neuron to the post-synaptic neuron. Synaptic weight, also known as synaptic connection and synaptic efficacy, stands for the amount of influence one neuron has on another. The majority of the development, memory, and learning in neural networks are attributed to synaptic plasticity, which refers to activity-dependent modifications of synaptic weights [11–13]. In a neuromorphic system, synaptic weight can correspond to the amplitude or strength of a connection between two nodes, in other words, the conductance of artificial synaptic elements [14].

Artificial synaptic devices associated with various materials and working mechanisms have been extensively studied in recent years [15–18]. In particular, vertical two-terminal memristive devices including electrochemical metallization memory (ECM) [19, 20], valence change memory (VCM) [21, 22], and phase-change memory (PCM) [23, 24] are regarded as probable candidates for artificial synapses owing to their simplicity of fabrication and extensibility of structural integration as crossbar arrays [25–27]. Nevertheless, these conventional memristive synaptic devices have intrinsic difficulties in precise weight control due to their random nature, resulting in nonlinear and asymmetric weight updates that significantly degrade the performance of artificial neural networks. Hence, their practical application as synaptic elements in hardware neuromorphic systems is severely restricted. Whereas, three-terminal synaptic transistors have attracted substantial interest due to reliable and notable synaptic characteristics [28–31]. Employing completely independent terminals for programming (gate) and reading (drain) facilitates linear and less distributed weight control operation. Recently, three-terminal synaptic devices associated with the electrochemical reactions of Li ions have been discovered to have improved synaptic properties [32–36]. Li ions diffuse gradually from the matrix in response to external stimuli, ensuring high controllability in plasticity modification. In addition, the migrations of Li^+^ ions do not induce considerable structural deformation upon intercalation and deintercalation, allowing stable and reversible operation. Synaptic transistors, despite their remarkable ability to perform linear and noise-free weight updates, have fundamental limitations in the realization of hardware neuromorphic systems. The three-terminal configurations with large and complex structures impede the crossbar array implementation required for high-density integration. There are some studies have reported synaptic operation involving ionic diffusion of Li cations in vertical two-terminal configuration [37–40]. Still, their capabilities as artificial synaptic devices remained unsatisfactory. Major synaptic functionalities including basic plasticity were absent, and weight updates were nonlinear, asymmetric, and fairly scattered. Furthermore, since neural networks incorporating synaptic devices had not been modeled, the effectiveness of hardware neuromorphic systems was not investigated. To be considered a viable synaptic device, thorough evaluations of the overall synaptic properties and performance simulation on artificial neural networks should be accomplished.

In this work, we propose a novel vertical Au/Li_*x*_CoO_2_/Pt device with excellent synaptic performance, demonstrating the feasibility of artificial synapses based on Li-ion intercalation. The synaptic behaviors were successfully attained by gradually depleting the Li ions in the Li_*x*_CoO_2_ thin film via controlled migration of Li ions within the film. The amount of Li ions inserted and extracted upon the matrix could be exactly regulated by the weight control spike, allowing for enhanced weight controllability over conventional two-terminal memristors. The reversible synaptic operation enables stable and linear weight updates. Bio-plausible synaptic characteristics such as short-term plasticity (STP) and long-term plasticity (LTP), paired-pulse facilitation (PPF), and spike-timing-dependent-plasticity (STDP) have been successfully imitated. The obtained long-term potentiation (LTP) and long-term depression (LTD) curves are discussed in symmetricity, nonlinearity, and dynamic range, which have a significant impact on the performance of the artificial neural networks. Moreover, investigations on Li ion migrations in Au/Li_*x*_CoO_2_/Pt artificial synapses clearly revealed the weight control mechanism. Finally, several types of artificial neural networks were grafted to simulate the performance of Li_*x*_CoO_2_-based neuromorphic systems. The deep convolutional neural networks (CNNs) were introduced to evaluate the reliability of analog computing which harnessed the synaptic weights of Li_*x*_CoO_2_ artificial synapses. The image inference reflecting the programming noise of the weight updates was executed based on the experimental LTP/LTD data. Likewise, the learning capabilities of a crossbar array constructed of Li_*x*_CoO_2_ artificial synapses were estimated through deep neural networks (DNNs). Consequently, the Li_*x*_CoO_2_-based neural networks outperformed three-terminal synaptic transistors in inference accuracy, demonstrating the potential of dependable hardware operations for neuromorphic computing.

## Experimental Section

### Target Preparation

The Li excess Li_1+*x*_CoO_2_ targets were prepared by sintering a mixture of high purity LiCoO_2_ (Sigma-Aldrich) and 10% excess Li_2_O (Alfa Aesar) powders to compensate for Li loss during deposition. The mixed powders were ball milled for 72 h and dried at 80 °C for 2 h. The targets pressed into 1-inch diameter were sintered at 400 °C for 2 h and 1000 °C for 10 h.

### Device Fabrication

The 80 nm Pt bottom electrodes were coted on SiO_2_/Si substrates with a Ti adhesive layer of 20 nm via electron beam evaporation. The Pt/Ti/SiO_2_/Si substrates were cleaned successively in acetone, isopropanol, and deionized water under ultrasonication. LiCoO_2_ thin film was deposited on Pt/Ti/SiO_2_/Si substrates by The Pulsed laser deposition (PLD) technique. The Li_1+*x*_CoO_2_ target and substrate were placed inside a vacuum chamber of the PLD with a pressure of 1 × 10^−6^ Torr. The target–substrate distance was kept at 50 mm. A KrF excimer laser (COMPLEX PRO 201F, COHERENT) with wavelength of 248 nm was used for the deposition. Laser fluence was controlled at 2.5 J cm^−2^ and a repetition rate at 5 Hz. Film deposition was carried out with 200 mTorr oxygen partial pressure at 300 °C. As a final procedure, Au top electrodes with a size of 50 μm × 50 μm were deposited on the LiCoO_2_ film by electron beam evaporation using a shadow mask under a pressure of 1 × 10^−6^ Torr at room temperature.

### Device Characterization

The X-ray diffraction (XRD) measurements were conducted using an X-ray diffractometer (Bruker Miller Co., D8-Advance) with Cu Kα radiation (*λ* = 1.54056 Å). XRD data were measured at room temperature in the 2θ range of 10°–80° with a step size of 0.02° and a scan speed of 3° min^−1^. The LiCoO_2_ thin film surface and cross sectional images of the device were obtained using a field emission scanning electron microscope (ZEISS, MERLIN Compact) with an in-lens secondary electron detector at a 5 kV accelerating voltage. The topography of LiCoO_2_ films deposited on an Pt/Ti/SiO_2_/Si substrates were estimated using an AFM (Park systems, XE-100). The elemental depth profile analysis was performed using ToF–SIMS (ToF.SIMS-5, IONTOF) with Bi^+^ primary source and Cs^+^ etching source. The etching area is 200 μm× 200 μm and the analysis area is 50 μm × 50 μm. The XPS (PHI, Versaprobe III) was used to analyze the chemical bonding of Li_*x*_CoO_2_ film. The Raman spectra were obtained by Laboratory RAM HR (Horiba Jobin Yvon, Japan) with excitation wavelength of 532 nm. The electrical properties of the device were measured using a Keithley 2636A.

## Results and Discussion

### Artificial Two-Terminal Ionic Synapse

**Fig. 1:**
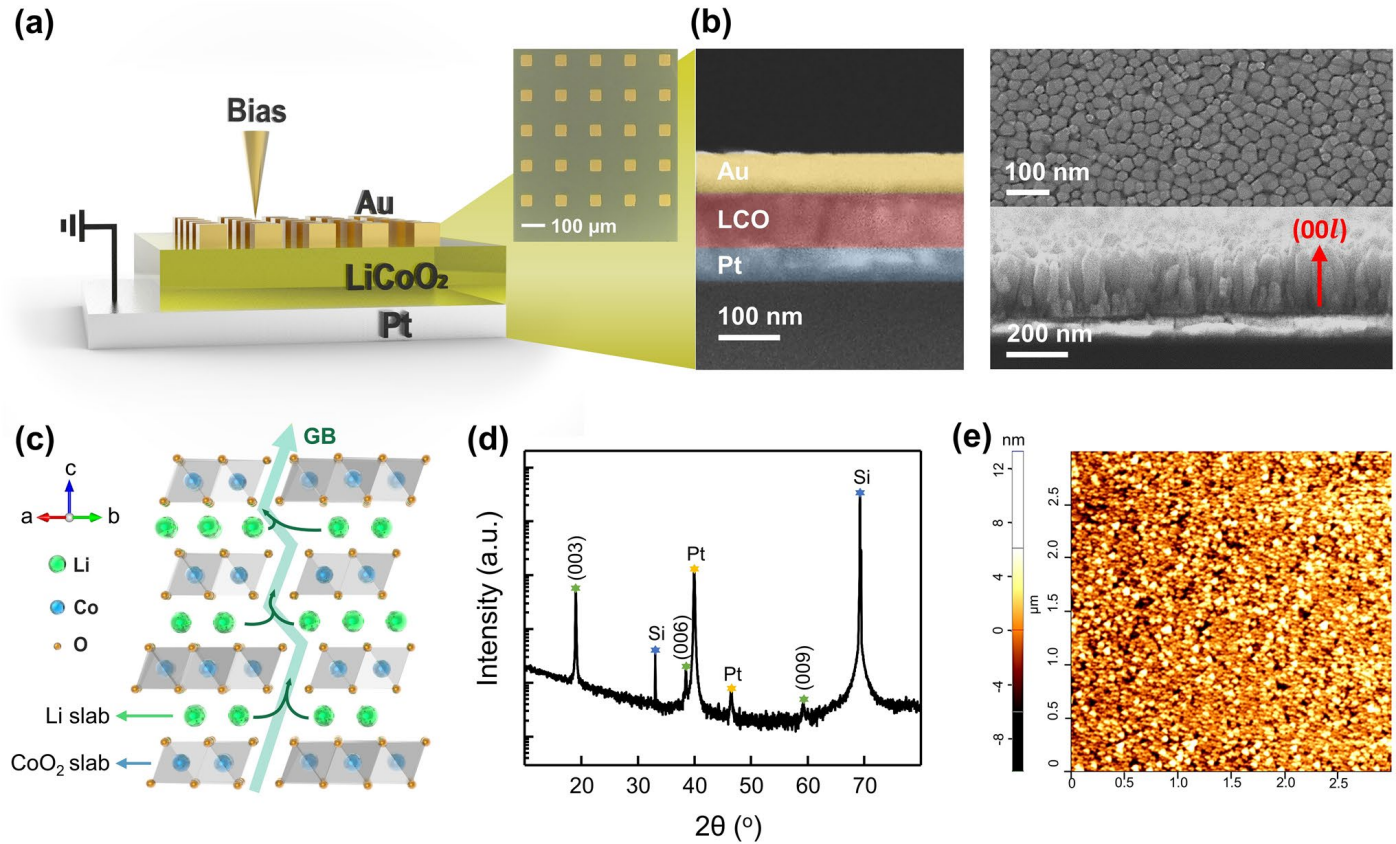
Device structure and characterization of LiCoO_2_ thin film. **a** Schematic structure and optical microscope image of an Au (TE)/LiCoO_2_/Pt (BE) vertical two-terminal artificial synapse. **b** Color-coded SEM image of vertically stacked Au (TE)/LiCoO_2_/Pt (BE)/SiO_2_/Si device. The top-view and cross sectional SEM image of (001) textured LiCoO_2_ thin film. **c** Crystal structure of LiCoO_2_. **d** XRD patterns of (001)-oriented LiCoO_2_ thin film. **e** AFM image of the LiCoO_2_ thin film. (RMS = 2.2 nm)

**Fig. 2:**
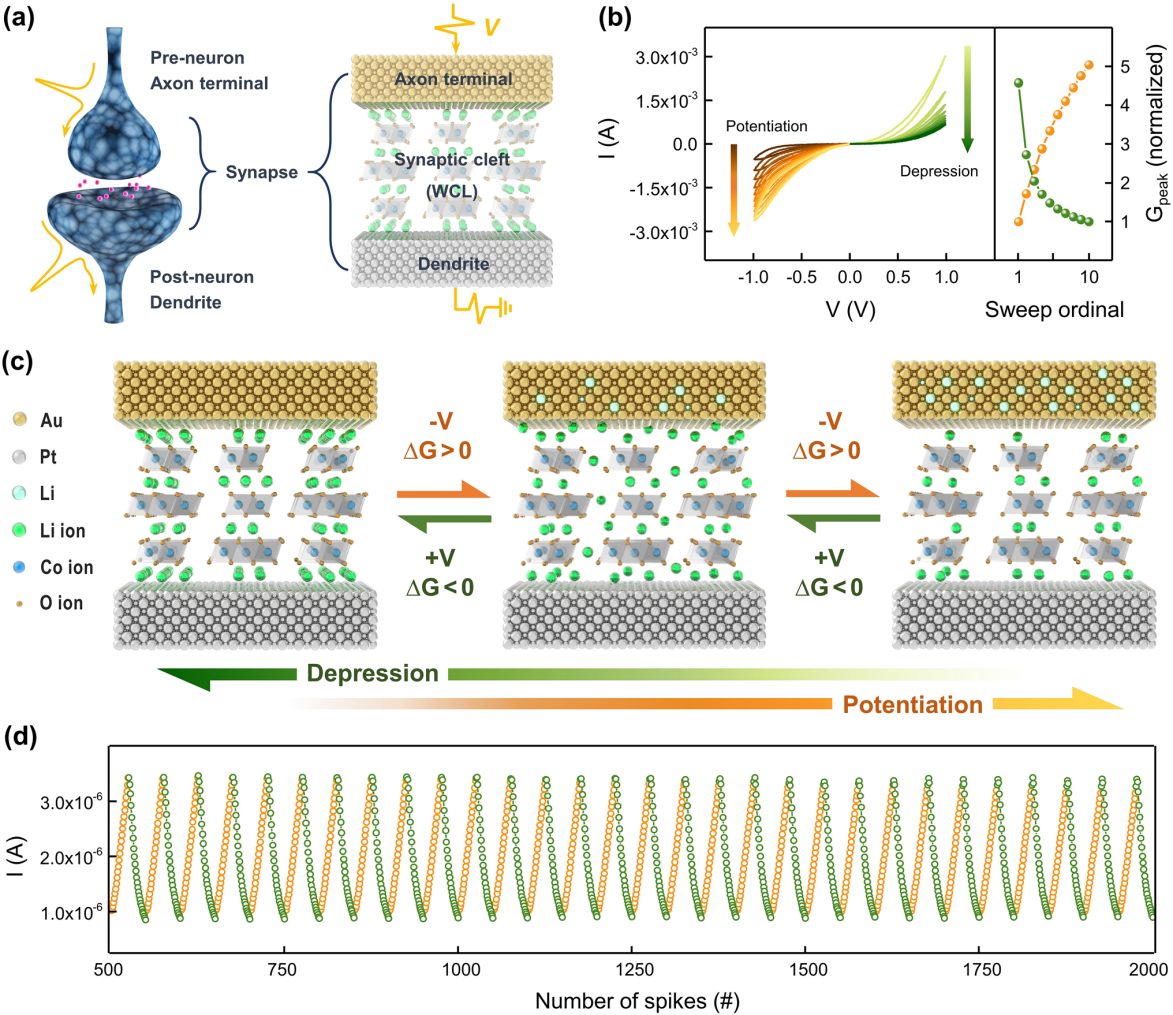
The fundament of synaptic weight modulation of the Au/Li_*x*_CoO_2_/Pt artificial synapse. **a** A graphic depicting the artificial synapse that imitates biological synapses. **b** The potentiation (depression) of synaptic weights according to 10 successive negative (positive) DC voltage sweeps and the increase (decrease) ratio in peak conductance. **c** Schematic illustration of the weight control mechanism in Au/Li_*x*_CoO_2_/Pt synaptic device. **d** Spike-induced synaptic potentiation and depression with sequential input of 25 negative and positive spikes each per cycle

It has been proved that LiCoO_2_, one of the most traditional cathode materials for secondary lithium-ion batteries, provides stable intercalation and deintercalation of Li ions with high reversibility [41–44]. In the non-stoichiometric Li_*x*_CoO_2_ (*x* < 1), the electrical conductivity tends to increase as Li is deficient [45–47]. The conductance tunability with respect to the Li content in matrix allows its application as a weight control layer for artificial synapses. Here, vertically stacked Au/Li_*x*_CoO_2_/Pt artificial synapses were fabricated with the advantages of process simplicity and structural effectiveness as well as reliable synaptic operation. Figure 1a displays the schematic structure and optical microscope image of a two-terminal Au (TE)/LiCoO_2_/Pt (BE) artificial synapse. Pulsed laser deposition (PLD) was used to deposit a stoichiometric LiCoO_2_ film of 100 nm thickness on the Pt bottom electrode. More information on device fabrication can be found in the materials and methods section. The scanning electron microscope (SEM) image in Fig. 1b showed the columnar growth of hexagonal-faceted LiCoO_2_ grains with an average size of 20 nm. LiCoO_2_ belonging to space group R3m has a layered structure with hexagonal symmetry. As illustrated in Fig. 1c, a hexagonal lattice consists of close-packed arrangements of oxygen ions with alternating layers of lithium and cobalt ions occupying octahedral sites. The CoO_6_ octahedra share edges to form CoO_2_ slabs, and anisotropic diffusion of lithium ions within the grains occurs transversely along the slabs perpendicular to the *c*-axis [48, 49]. The vertical transport of Li ions parallel to the *c*-axis is carried out via grain boundaries and does not penetrate the CoO_2_ slabs [50, 51]. Therefore, the highly (0 0 3) oriented LiCoO_2_ thin film with fine grain size, as confirmed in the SEM image (Fig. 1b) and X-ray diffraction patterns (Fig. 1d), minimized the diffusion path of Li ions. As a result, rapid intercalation and deintercalation of lithium ions with low energy consumption were effectively achieved [52–54]. Further, the CoO_2_ textured out-of-plane surface of LiCoO_2_ prevented spontaneous re-insertion of extracted Li ions into the LiCoO_2_ matrix, thus, nonvolatile characteristics can be secured. The atomic force microscopy (AFM) analysis in Fig. 1e revealed that the PLD-deposited LiCoO_2_ thin film had a uniform and smooth surface, with a root mean square (RMS) roughness of 2.2 nm. Figure 2a depicts an artificial synapse in mimicking a biological synapse. Biological synapses transmit specific information-bearing neuronal spikes from pre-synaptic neurons to post-synaptic neurons depending on the strength of the synaptic connection. In the Au/Li_*x*_CoO_2_/Pt artificial synapse, the Li_*x*_CoO_2_ weight control layer changed the device conductance in response to external stimuli, imitating synaptic plasticity, a modification in synaptic connections. Structurally, the Au top electrode served as the pre-synaptic interface to the axon terminal, the Pt bottom electrode served as the post-synaptic interface to the dendrite, and the Li_*x*_CoO_2_ film served as the synaptic cleft, the junction gap between the axon terminal and dendrite. The synaptic characteristics were estimated by applying a pre-synaptic voltage to the Au top electrode while grounding the Pt bottom electrode. Figure 2b shows modification in synaptic efficacy along with successive DC voltage sweeps. During the potentiation process, the synaptic connection was gradually strengthened according to the consecutive 10 negative DC voltage sweeps in clockwise (0 V →  −1 V → 0 V). Contrary, it was reduced back after 10 positive DC voltage sweeps in counterclockwise (0 V → 1 V → 0 V), exhibiting synaptic depression behavior. The peak conductance of each sweep implies that the device conductance increased and decreased by a factor of 5, respectively, as the sweep progressed. The DC sweeps were conducted at a rate of 0.5 V s^−1^ throughout the synaptic modulation. Figure 2c displays a schematic illustration of the weight control mechanism of the Au/Li_*x*_CoO_2_/Pt artificial synapses. When the negative voltage was applied to the top electrode, Li cations were deintercalated from Li_*x*_CoO_2_ lattice and inserted into the Au film [55, 56], increasing the conductance of the delithiated Li_*x*_CoO_2_ layer. Conversely, the applied positive voltage led the Li cations to be extracted back from the lithiated Au electrode and reintercalated into the Li_*x*_CoO_2_, restoring the film conductance. Hence, with appropriate weight control activity regulating the Li content in Li_*x*_CoO_2_ films, it is possible to emulate synaptic properties based on synaptic weight potentiation and depression. During the lithiation–delithiation process, the Au film underwent solid solution reactions with Li to form LiAu_*x*_, with little structural change [55, 57]. Figure S1 depicts the analog and spike-induced synaptic properties of Li_*x*_CoO_2_-based artificial synapses with different top electrodes. In a high dynamic range of linear weight modulation, the Au electrode provides the most stable and reversible insertion and extraction of large amounts of Li. Figure S2 displays the sweep rate dependency related to ion dynamics in analog weight modification of Au/Li_*x*_CoO_2_/Pt artificial synapse. As the sweep rate was lowered from 20 to 0.5 V s^−1^, more Li ions migrated causing larger hysteresis. The spike-induced synaptic potentiation and depression were demonstrated, as shown in Fig. 2d. To adjust the synaptic connections, 25 negative spikes of − 1.5 V with 10 ms and positive spikes of 1.1 V with 10 ms were sequentially input per cycle. Following each active pulse, 0.1 V read pulses were used to measure the synaptic connectivity. A series of negative voltage spikes resulted in four-fold weight potentiation and following positive voltage spikes caused weight depression to its original state. Figure S3 shows the endurance of the Li_*x*_CoO_2_ artificial synapses to spike-induced weight modulation. For 350 cycles, updates of synaptic weights were accomplished by sequential input of 25 negative and positive spikes per cycle. During 17,500 times of weight updates, the device exhibited outstanding cyclability and endurance. After 350 cycles, it is about 5% more conducting than the initial state. This is presumed to be owing to a little quantity of residual Li accumulating inside the Au film, while the weight updates were repeated. The endurance constraints become apparent when the devices are employed for in situ training, hence a substantial improvement in endurance is necessary for workable neuromorphic applications. Table S1 lists the weight modulation parameters of artificial synaptic devices with various materials and mechanisms. The Au/Li_*x*_CoO_2_/Pt artificial synapse showed a relatively low operation voltage and fast response speed due to the facile Li-ion migration. The excellent linearity of the lithium-mediated devices on weight updates suggests their suitability for artificial synapses. The estimation of nonlinearity is covered in further detail in the following section.

### Activity-Dependent Synaptic Plasticity

**Fig. 3:**
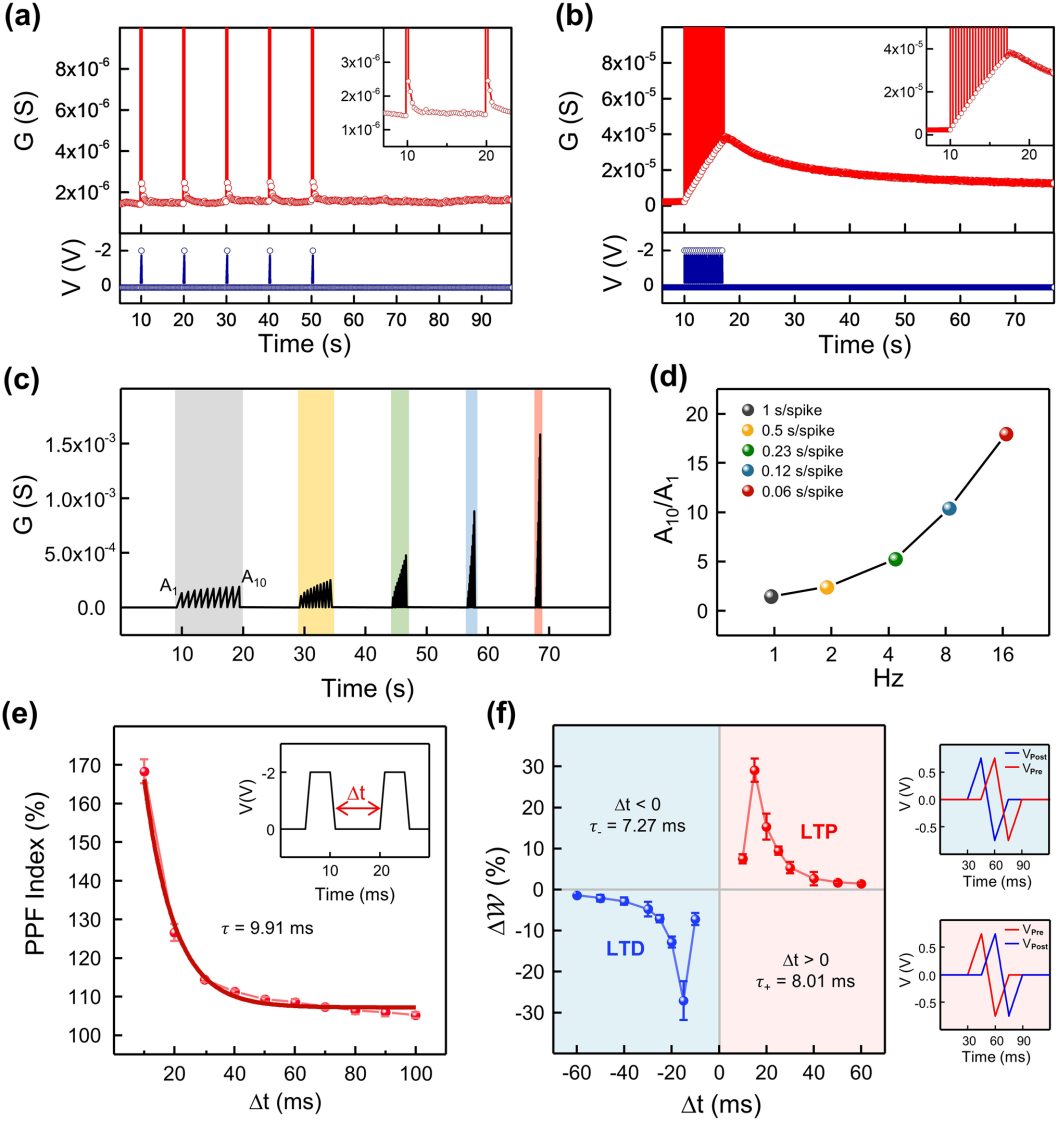
Synaptic plasticity for various spike intervals. **a** Short-term plasticity (STP) with spike intervals of 10 s. **b** Long-term plasticity (LTP) with spike intervals of 0.33 s. **c** Spike-rate-dependent-plasticity (SRDP) characteristics depending on different spiking rate and **d** conductance change ratio over 10 spikes. **e** Paired-pulse facilitation (PPF) index as a function of interval time between two spikes. **f** Spike-timing-dependent-plasticity (STDP) in the form of asymmetric Hebbian learning rule

Synaptic plasticity is a phenomenon in which a specific pattern of synaptic activity results in a change in synaptic weight [11]. Synaptic plasticity can be classified into two types in terms of memory retention: short-term plasticity (STP) and long-term plasticity (LTP) [12]. Short-term plasticity refers to a transient deformation in synaptic efficacy that leads to the loss of neuronal information within seconds in general. STP is normally triggered by brief bursts of stimulus, whereas LTP is elicited by rapid repetition of stimuli. Long-term plasticity is a long-lasting change in synaptic connectivity where high-frequency stimulation affects the efficacy of subsequent synaptic transmission, exceeding the temporal limitation of STP. In Fig. 3a, a demonstration of STP showed momentary reinforcement in synaptic weight that promptly dissipated afterward. A single stimulus (− 2 V, 50 ms) applied with an interval of 10 s provoked a short-lasting potentiation. The synaptic weight returned to its initial state within 3 s, having no effect on the ensuing synaptic weight. In contrast, as seen in Fig. 3b, repetitive activation by frequent stimuli led to long-term potentiation in synaptic weight that was maintained over 70 s. Spikes with the identical conditions as in STP (− 2 V, 50 ms) but with a much shorter interval of 0.33 s were utilized for LTP modulation. After 20 activations, the synaptic weight enhanced 9.8 times compared to the pristine, and after a slight decay, the potentiation ratio converged to 3.5 times. These activities are analogous to human memory control procedures hypothesized by Atkinson and Shiffrin [58]. The transition from STP to LTP is thought to be caused by a sufficient supply of energy from multiple stimuli. In the case of a sporadic stimulus, Li ions accumulated and then dispersed at the interface between the Li_*x*_CoO_2_ film and the Au electrode rather than penetrating the Au film. Repeated stimuli, on the other hand, allowed the accumulated Li ions to be injected into the Au electrode, facilitating nonvolatile weight adaptation. Figures S4 and S5 display multistate LTP retention and temperature-dependent retention characteristics for 1000 s. The spike-rate-dependent-plasticity (SRDP) characteristic in Fig. 3c implied that the higher the frequency of the weight control spikes, the stronger the synaptic potentiation. Ten weight control spikes of − 2 V and 30 ms were delivered at five different frequencies ranging from 1 to 0.06 s/spike. The conductance change ratios for 10 spikes rise exponentially with shorter input signal intervals, as seen in Fig. 3d. Paired-pulse facilitation (PPF), a form of STP, is a neurological phenomenon observed when two close spikes are triggered rapidly [59]. The transmission of the second neural signal is amplified since the immediately preceding impulse induces an increase in the synaptic connection [60]. Figure 3e depicts the PPF index expressed as a function of the spike interval Δ*t*. The PPF index is defined as the ratio of the amplitude of the second response (A2) to the first response (A1):

1 $${\text{PPF}}\;{\text{ratio}} = \left( {A_{2} - A_{1} } \right)/A_{1}$$

A pair of stimuli of − 2 V and 5 ms were supplied at intervals ranging from 10 to 100 ms. The PPF ratio peaked at 170% when Δ*t* was 10 ms and declined exponentially toward 100% with a time constant of 9.91 ms as the inter-spike delay grew. Spike-timing-dependent plasticity (STDP) is a weight specialization based on a firing order provoked by close temporal interactions between the spiking of pre-and post-synaptic neurons [13]. The STDP characteristic was successfully demonstrated by supplying weight control spikes to the top and bottom electrodes with a chronological difference. Figure 3f represents a Hebbian STDP in which pairs of pre-leading-post and post-leading-pre spikes with intervals of tens of milliseconds result in long-term potentiation (LTP) and long-term depression (LTD), respectively [61–63]. LTP is induced when pre-synaptic spikes fire before postsynaptic spikes and the spiking time difference Δ*t* is positive. Likewise, LTD occurs in the reverse order, with post-synaptic spikes preceding pre-synaptic spikes, with a negative Δ*t*. The degree of synaptic potentiation tended to diminish as ∆t receded in a positive direction, and vice versa for depression. The plotted ∆*w*, the change in synaptic weight, can be expressed as an exponential decay function for ∆*t* as follows:

2 $$G \left( {\Delta t} \right) = \left\{ {\begin{array}{*{20}l} {A_{ + } \exp \left( { - \frac{\Delta t}{{\tau_{ + } }}} \right)} \hfill & {if \Delta t \ge 0} \hfill \\ { - A_{ - } \exp \left( { - \frac{\Delta t}{{\tau_{ - } }}} \right)} \hfill & {if \Delta t < 0} \hfill \\ \end{array} } \right.$$

The extracted time constants of *τ*_+_ and *τ*_−_ were 8.01 and 7.27 ms, respectively, implying millisecond response rates comparable with biological synapses [61–64]. Both PPF and STDP characteristics were obtained statistically for 5 devices. In Li-based artificial two-terminal ionic synapses, bio-plausible synaptic behaviors were successfully implemented by applying spikes at different intervals.

**Fig. 4:**
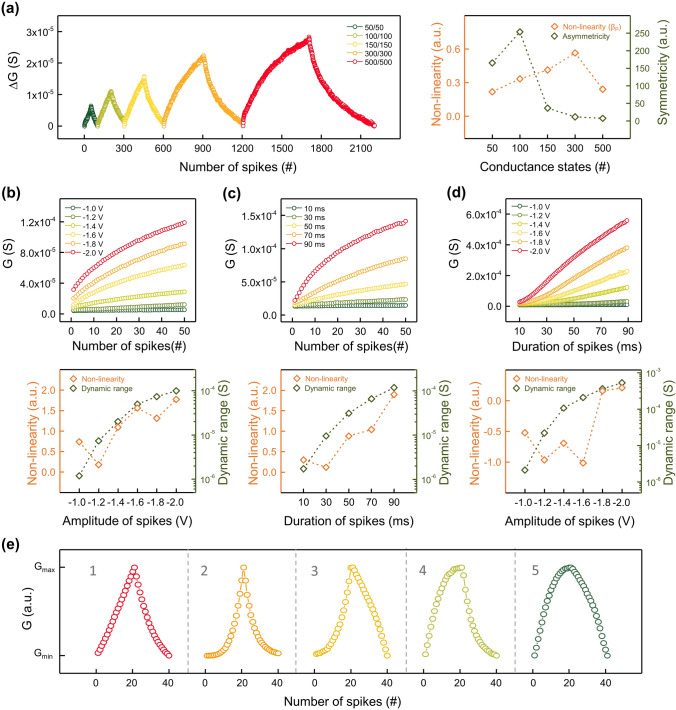
Synaptic plasticity under various spike conditions. Diverse LTP/LTD curves and extracted symmetricities, nonlinearities, and dynamic ranges obtained under different weight control regimes. **a** Spike-number-dependent-plasticity (SNDP) characteristics depending on different numbers of weight control spikes. **b** Spike-voltage-dependent-plasticity (SVDP) characteristics depending on different amplitudes of weight control spikes. **c** Spike-width-dependent-plasticity (SWDP) characteristics depending on different durations of weight control spikes. **d** Spike-voltage-width-dependent-plasticity (SVWDP) characteristics depending on different amplitudes and durations of weight control spikes. **e** LTP/LTD curves customized in different shapes using spike tailoring

The activity-dependent LTP/LTD properties of Au/Li_*x*_CoO_2_/Pt artificial synapses were further explored under different weight control regimes. The evaluations of nonlinearity, symmetricity, and dynamic range, which have a substantial impact on the performance of neuromorphic computing, were also accompanied [7, 10]. Nonlinearity refers to the deviation from the ideal update of synaptic weights where the step size remains constant. The weight update relations with nonlinearity parameters were covered in Note S1. Figure S6 depicts LTP/LTD curves with varying curvatures depending on nonlinearity. Symmetricity stands for the degree to which the traces of individual weight levels coincide in the weight change trajectories of potentiation and depression. Note S2 and Fig. S7 contains details of symmetricity determination in LTP/LTD curves. Dynamic range is defined as the scope of available device conductance where the distinct synaptic weights can be assigned during computation in neural networks, i.e., (*G*_max_ − *G*_min_). Figure 4a exhibits spike-number-dependent-plasticity (SNDP). Weight control spikes (∓ 2 V, 20 ms) were administered 50 to 500 times for potentiation and depression, respectively. The nonlinearity and symmetricity values were derived from the LTP/LTD profiles. The LTP curves of SNDP offered excellent nonlinearity (*β*_P_) of fairly low values, which tended to increase somewhat as the number of conductance states grew. Besides, the weight updates of 100 or less showed reliable symmetricity with high extracted values. Figure S8 provides extra estimations of the nonlinearity of LTD curves (*β*_D_) and dynamic range. In addition to the number of spikes, other activity patterns related to spike amplitude and spike duration were also introduced. In Fig. 4b, the spike-voltage-dependent-plasticity (SVDP) was validated in 0.2 V increments from − 1 to − 2 V for 50 ms duration. Likewise, spike-width-dependent-plasticity (SWDP) was examined at 20 ms intervals from 10 to 90 ms at − 1.5 V, as presented in Fig. 4c. In both SVDP and SWDP, the greater the energy of the weight control spikes, the worse the linearity and the drastic expansion of the dynamic ranges. This is because the weight updates for relatively low energy stimuli strengthened the synaptic weights in a narrow dynamic range where saturation of Li in the Au electrode did not occur. Moreover, the spike-voltage-width-dependent-plasticity (SVWDP), a combination of amplitude and duration modulation, was presented in Fig. 4d. At a given voltage, the spike duration steadily rose from 10 to 90 ms, boosting the energy of the weight control spikes in progression. Thus, the weights can be updated linearly without saturation by continuously supplying adequate activation energy. Figure S9 displays the ratio of the minimum conductance measured in the initial state to the maximum conductance obtained after potentiation in SVDP, SWDP, and SVWDP. The stronger the energy of the spikes, the greater the on/off ratio of the devices. Lithium-mediated artificial ionic synapses elaborately alter the synaptic connections into certain forms in response to external stimuli. The superb weight tunability of Au/Li_*x*_CoO_2_/Pt artificial synapses was demonstrated by customizing the weight update profiles via spike pattern tailoring. Figure 4e displays custom LTP/LTD curves of five different shapes. Depending on the specific activity pattern of the weight control spikes, the weights change abruptly or slightly and determine the morphology of the entire curves. Figure S10 and Table S2 present the custom patterns of spike trains used for LTP/LTD tailoring. Selecting an appropriate spike condition can afford acceptable weight updates practically usable in neuromorphic computing with reasonable nonlinearity, symmetricity, and dynamic range. Table 1 compares the overall synaptic performance of Li-based artificial synaptic devices and two-terminal Li_*x*_CoO_2_-based artificial synapses. The Au/Li_*x*_CoO_2_/Pt devices showed improved nonlinearity (*α*) and asymmetry ratio owing to the reversible synaptic operation obtained by structural optimization of the weight control layer. Furthermore, various types of synaptic plasticity were demonstrated for the first time in Li ion-based synaptic devices, and each synaptic behavior was intensively explored. The analysis of nonlinearity (*α*) and asymmetric ratio for LTP/LTD curves are provided in Note S1 and Note S3.

**Table 1:** Performance comparison of synaptic properties of recently reported Li^+^ ion based artificial synapse device

Device Type	Structure		Non linearity [*α*]^1^	Asymmetric ratio^2^	Implemented synaptic plasticity	Learning accuracy (error)^3^	Refs.
	Channel	Electrolyte					
3T Synaptic Transistor	WSe_2_	LiClO_4_	1.7/0.4	0.19	Pulse PnD, STP/LTP, PPF, SWDP, SRDP	N/A	[32]
	MoO_3_	LiClO_4_	2.6/− 0.4	0.31	Pulse PnD, PPF SVDP, SWDP	87.3% (10%)	[33]
	LiCoO_2_	LiPON	0.7/0.8	0.17	Pulse PnD	97.8% (0.4%)	[34]
	LiCoO_2_	Li_3_PO_*x*_Se_*x*_	1.33/− 0.34	0.12	Pulse PnD	91.0% (6.1%)	[35]
	WO3	LiClO4	0.96/-0.11	0.26	Pulse PnD, SVDP	93.3% (3.7%)	[36]
2T Vertical Memristor	Au/Li_*x*_CoO_2_/SiO_2_/TiO_2_/p^++^-Si		N/A	N/A	Analog PnD, Pulse PnD, STDP, SRDP	N/A	[37]
	Ni/Li_*x*_CoO_2_/a-Si		N/A	N/A	Analog PnD, Pulse PnD, STDP	N/A	[38]
	Cr/LiCoO_2_/a-Si/TiN		N/A	N/A	Pulse PnD, LTP	N/A	[39]
	Pt/LiSiO_*x*_/TiN		N/A	N/A	Analog PnD, STP/LTP, STDP, PPF	N/A	[40]
	Au/Li_*x*_CoO_2_/Pt		0.83/0.46	0.09	Analog PnD, Pulse PnD, STP/LTP, SRDP, PPF, STDP, SVDP, SWDP, SVWDP	95.36% (0.14%)	**This work**

j543[{^1^The nonlinearity (*α*) is explained in Fig. S6 and Note S1

^2^The asymmetric ratio is described in Note S3

^3^The error represents the difference between the theoretical accuracy and the simulated accuracy of the device

### Li ion Transfer Investigation

**Fig. 5:**
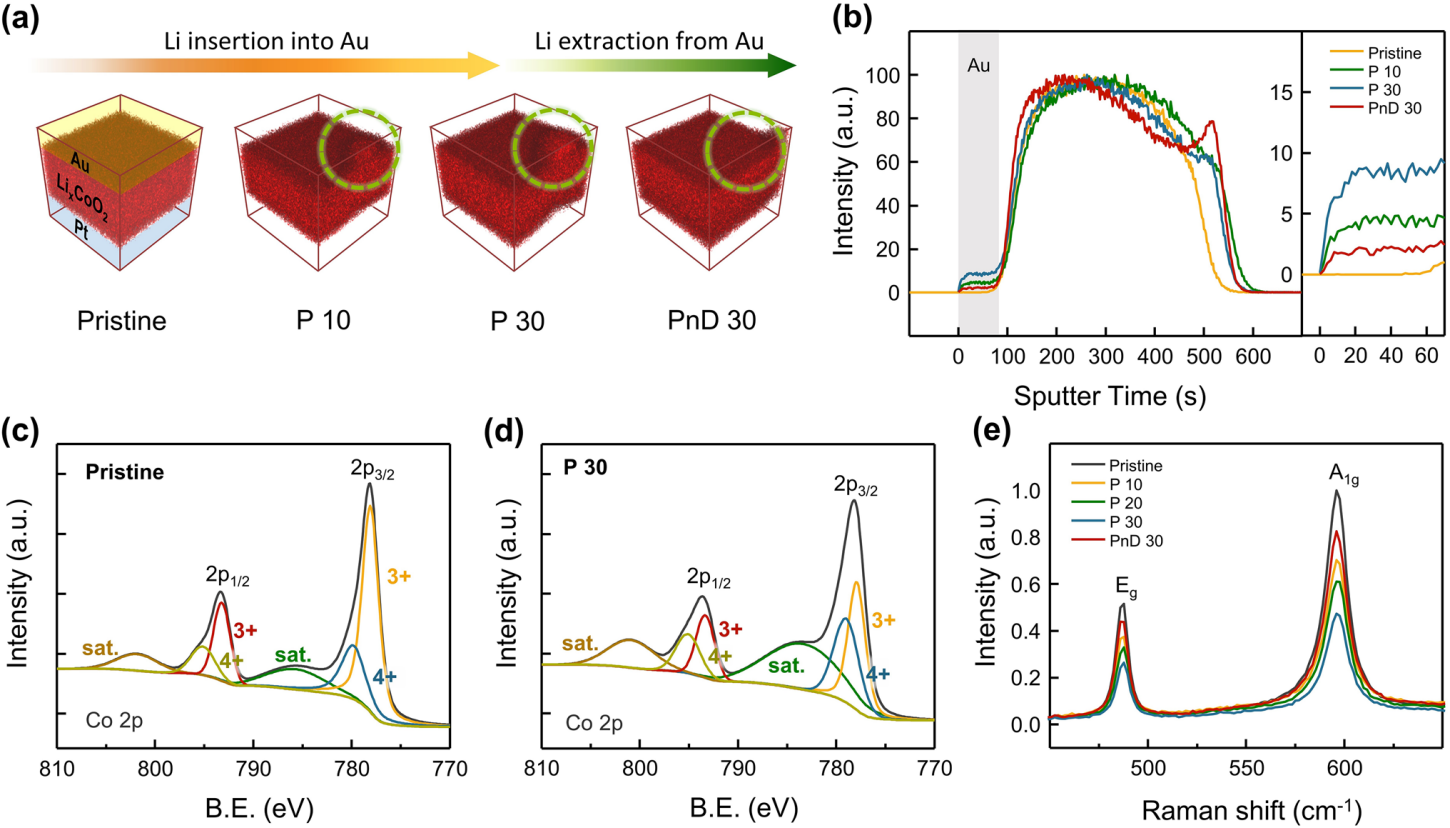
Intercalation and deintercalation of Li ions in Au/Li_*x*_CoO_2_/Pt artificial synapse. **a**, **b** ToF–SIMS 3D mappings and depth profiles of Li ions in Au/Li_*x*_CoO_2_/Pt structure at four different synaptic weight states. The amount of Li ions in the Au top electrode increased as negative sweeps (potentiation) were performed from the initial state to 30 times, and decreased after 30 times of positive sweeps (depression). **c**, **d** XPS spectra of the Co 2p region for Li_x_CoO_2_ film in a pristine state and after 30 times potentiation. The ratio of Co^4+^/Co^3+^ grew since the Co cations were oxidized from Co^3+^ to Co^4+^ as the Li^+^ cations were deintercalated from the Li_*x*_CoO_2_ framework. **e** Raman spectra for Li_*x*_CoO_2_ thin film. The relative intensity reduced as the Li_*x*_CoO_2_ became Li-deficient due to deintercalation of Li ions, and recovered with the re-intercalation of Li ions

Next, Li ion transfer in Au/Li_*x*_CoO_2_/Pt artificial synapses was investigated to provide the rationales for the aforementioned weight control mechanism. Each analysis was carried out at several distinct synaptic weight states: pristine, after 10/20/30 potentiations, and after 30 depressions following 30 potentiations. The *I–V* characteristics of analog voltage sweep-induced synaptic potentiation and depression performed for the analyses below are shown in Fig. S11. The time-of-flight secondary ion mass spectrometry (ToF–SIMS) proposed definite insight into the Li-ion insertion in the Au electrode. The 3D mapping images of Li ions in Au/Li_*x*_CoO_2_/Pt structure for the four synaptic activities are illustrated in Fig. 5a. In the initial state, the Li ions, depicted as red dots, resided only within the Li_*x*_CoO_2_ film, indicating a clear separation from the electrodes. As the negative sweeps were repeated up to 30 times during the potentiation process, the Li ions were gradually implanted into the Au top electrode to strengthen the synaptic weight. They were subsequently intercalated back into the Li_*x*_CoO_2_ lattice over the span of 30 positive sweeps, resulting in long-term depression. No residual Li in the Au top electrode after depression implies that the Li migration between the LiCoO_2_ matrix and Au thin film is a highly reversible process. The depth profiles of Li ions in Fig. 5b exhibited that the amount of Li reserved in the Au electrode sequentially increased and decreased as the process progressed. Figure S12 indicates that the Li_*x*_CoO_2_ framework was securely maintained throughout weight modulation. The X-ray photoelectron spectroscopy (XPS) was adopted to discover the intercalation and deintercalation of Li ions inside Li_*x*_CoO_2_. Figure 5c, d is XPS spectra of the Co 2*p* region for Li_*x*_CoO_2_ film in a pristine state and after 30 times potentiation, respectively. The Co 2*p* spectrum split into Co 2*p*_1/2_ and Co 2*p*_3/2_ peaks, each observed at 778.2 and 793.4 eV [65, 66]. The ratio of Co^4+^ to Co^3+^ grew since the Co cations were partially oxidized from trivalent to tetravalent as the Li^+^ cations were deintercalated from Li_*x*_CoO_2_ lattice [43, 44, 66]. The additional Co 2*p* profiles for after 10 potentiations, 20 potentiations, and 30 depressions are provided in Fig. S13. Besides, the area ratios of Co^4+^/Co^3+^ redox couple to five different weight control operations are available, where the fraction of the tetravalent varied in proportion to synaptic weight. The synaptic potentiation and depression were indirectly confirmed through Raman spectroscopy on the Li_*x*_CoO_2_ layer. As displayed in Fig. 5e, Li_*x*_CoO_2_ film exhibited two Raman-active *A*_1g_ and *E*_g_ modes at 596 and 497 cm^−1^, respectively [67, 68]. The Raman scattering intensity declined as the Li_*x*_CoO_2_ became Li-deficient due to the deintercalation of Li ions, and recovered again with the re-intercalation of Li ions. The decrease in relative intensity occurred due to an increase in electrical conductivity that lowers the optical skin depth of the incoming laser beam [68]. Therefore, the changes in Raman intensity can be qualitatively interpreted as a result of synaptic plasticity. The intercalation and deintercalation of Li ions proceeded reversibly without phase change of Li_*x*_CoO_2_ which generates extra peaks in the Raman band.

### Image Inference in Li_*x*_CoO_2_-Based Neuromorphic Systems

**Fig. 6:**
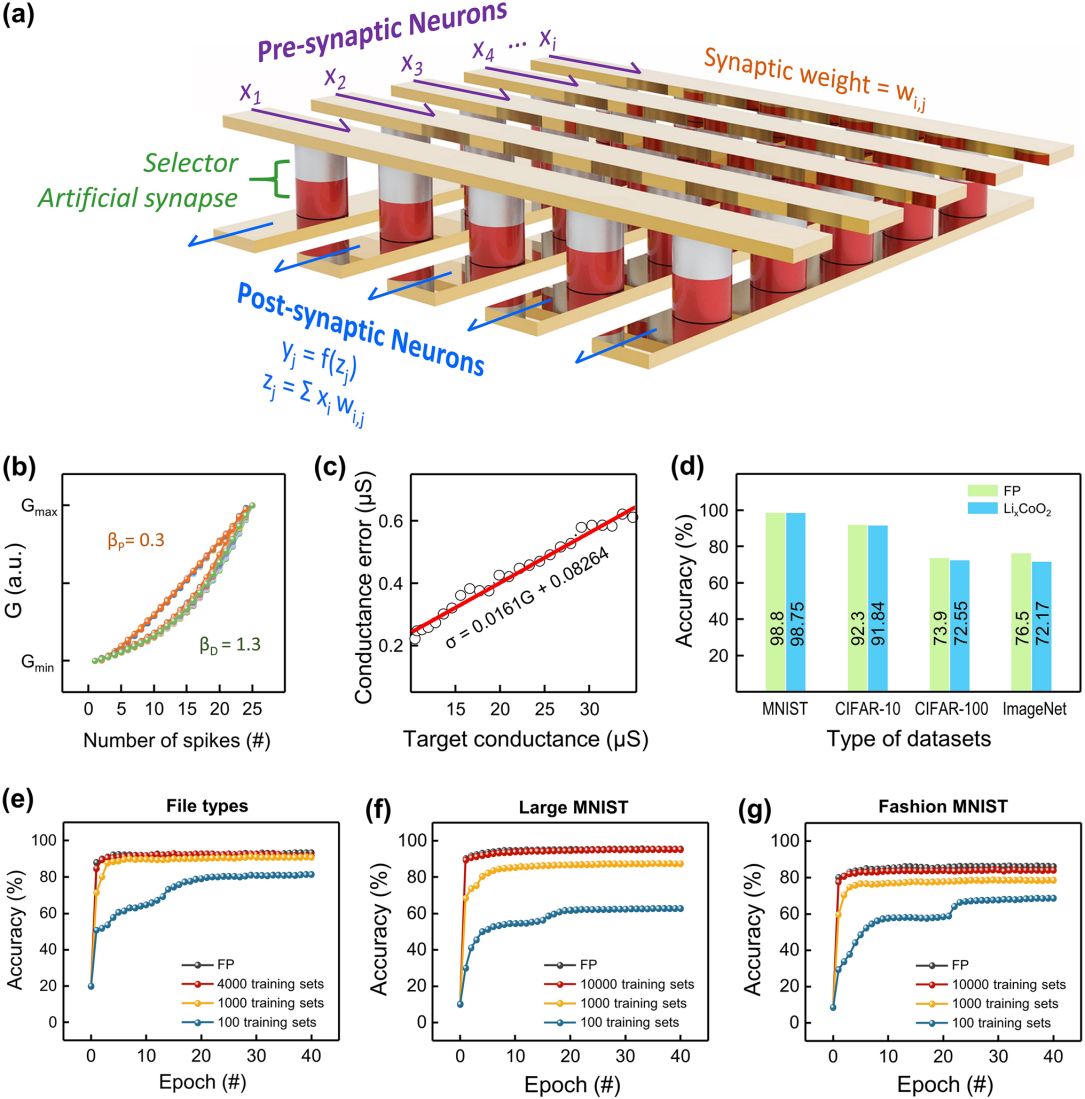
Image recognition of Li_*x*_CoO_2_-based neuromorphic systems. **a** Schematic illustration of the crossbar array structure for the neuromorphic circuit. **b** Cycle-to-cycle synaptic conductance for 30 cycles with fine nonlinearity. **c** Programming error characteristic of weight updates of Li_*x*_CoO_2_ artificial synapses over 30 cycles. **d** Inference accuracies on four types of datasets in floating-point and Li_*x*_CoO_2_-based CNNs. **e**–**g** Recognition accuracies of floating-point and Li_*x*_CoO_2_-based DNNs on the assigned training sets for File types, large MNIST, and fashion MNIST, respectively

All preceding investigations verified the exceptional synaptic properties of the Li-ion-mediated artificial synapses. To further verify the feasibility of the Li_*x*_CoO_2_-based synaptic array for hardware neuromorphic systems, image recognition employing artificial neural networks was conducted. Figure 6a presents a schematic illustration of a crossbar array structure that harnesses allocated synaptic connections to perform analog vector–matrix multiplication. The synaptic weights in the synaptic array are defined as the device conductance programmed by the input voltage signal supplied to the input neurons. The currents collected from the output neurons were processed through matrix multiplication with the input signal. The performance of artificial neural networks can be optimized through the learning process that programming synaptic devices to specific weights best suited for the data processing of a given algorithm. Since synaptic parameters such as nonlinearity, symmetry, and dynamic range are major determinants of weight targeting precision, obtaining desirable device properties is essential to achieving high neural network performance. Figure 6b shows superimposed 30 cycles of weight updates of Li_*x*_CoO_2_ artificial synapses acquired from the LTP/LTD data in Fig. 2d. The reliable synaptic operation was proven by low noise and fine nonlinearity of 0.3 for potentiation (*β*_P_) and 1.3 for depression (*β*_D_). The heat maps of Δ*G* versus *G* response during potentiation and depression are displayed in Fig. S14. The programming error characteristic of the device conductance is presented in Fig. 6c. The standard deviations were calculated for 60 weight programming for each target conductance of a total of 25 states. The low nonlinearity and programming error imply that the performance degradation caused by the physical limitations of the devices is not significant. Still, the weight relaxation over time, which might degrade performance in actual neural network applications, was not taken into account with programming error parameters. A non-negligible influence on learning outcomes is expected when relaxation effect is considered. The effects of programming errors in Li_*x*_CoO_2_ artificial synapses on computational achievement were examined through object classification based on convolutional neural networks (CNNs). A detailed description of the CNNs is covered in Note S4 and Fig. S15. The accuracy of image inference was compared between the floating-point (FP)-based CNNs and the Li_*x*_CoO_2_-based CNNs that have flaws in weight updates such as nonlinearity and noise. The Li_*x*_CoO_2_-based CNNs were operated by incorporating the device programming error *σ* = 0.0161*G* + 0.08264 into the weight allocation of the CNNs. Figure 6d schematizes the inference accuracies on four types of visual databases in FP-based CNNs and Li_*x*_CoO_2_-based CNNs. The entire inference processes for the MNIST [69], CIFAR-10 [70], CIFAR-100 [71], and ImageNet [72] databases were carried out using Crossbar Simulator provided by Sandia National Laboratories [73]. Table S3 tabulates the architecture of the CNN models used for image recognition, as well as the number of classes and input dimensions of the visual datasets. Utilizing floating-point as the synaptic weights to compute means that the attained accuracy is the theoretical maximum for the corresponding network architecture. In Li_*x*_CoO_2_-based CNNs, inferences on relatively small-dimensional datasets MNIST, CIFAR-10, and CIFAR-100 achieved high accuracy close to the theoretical performance of the networks. These impressive results are due to the low programming errors of Li_*x*_CoO_2_ artificial synapses.

Furthermore, multi-layer perceptrons (MLPs)-assisted learning simulation was implemented to assess the learning ability of Li_*x*_CoO_2_-based neuromorphic systems. The multistate weights measured in Fig. 2d were used as the source of synaptic connections for assignment to MLPs throughout the learning process. Figure S16 depicts the data processing flow in the MLPs [74]. Based on the given data, the DNN model was trained for up to 40 epochs, with each epoch exploring an optimal inferred model by training and testing on allocated training sets at random. In the training phase, stochastic gradient descent was used to optimize the synaptic connections of neural networks through a backpropagation-supervised learning algorithm [75]. The detailed structure and learning process of MLPs are covered in Note S5. As shown in Fig. 6d, Li_*x*_CoO_2_-based MLP performed admirably in file type identification, with only a 0.5% difference from FP. Figure 6f reveals that recognition accuracy on the MNIST datasets even rivaled FP after 40 epoch training with 10,000 training sets. Similarly, training on fashion MNIST achieved an accuracy of up to 84.5% for a theoretical maximum of 86.2% in Fig. 6g. Table 1 presents the inference accuracies on the MNIST datasets in MLPs simulations for the recently reported Li ion-based artificial synapses. Notably, the Li_*x*_CoO_2_-based artificial synapses outperformed the three-terminal synaptic transistors in terms of learning ability, with the least performance degradation for FP accuracy. These exceptional performances are owing to the highly linear and noise-free weight updates that enable precise and effective weight allocation during algorithmic learning.

## Conclusions

In this study, we implemented an artificial two-terminal ionic synapse utilizing intercalation and deintercalation of Li ions in Li_*x*_CoO_2_ film. The Au/Li_*x*_CoO_2_/Pt artificial synapses demonstrated synaptic potentiation and depression via weight control spikes associated with the progressive dearth of Li ions in Li_*x*_CoO_2_ films. The modification of synaptic connections was highly linear and uniform owing to the reversible Li^+^ migrations. Various synaptic behaviors, including bio-plausible synaptic plasticity, were successfully imitated for the first time in Li ion-based synaptic devices, accompanied by in-depth investigations. The Li_*x*_CoO_2_-based artificial synapses, in particular, exhibited impressive weight tunability based on activity-dependent synaptic plasticity. Under different weight control regimes, synaptic weights can be varied dynamically according to specific activity patterns to facilitate the control of synaptic properties. The evaluations of symmetricity, nonlinearity, and dynamic range of the LTP/LTD curves, which have a significant impact on the performance of neuromorphic computing, were also accompanied. High plasticity dependence on the activity patterns allows customization of the LTP/LTD profiles by tailoring weight control spikes. In addition, investigations on Li-ion migrations in Au/Li_*x*_CoO_2_/Pt artificial synapses provided the rationales for the weight control mechanism of the device. Lastly, the feasibility of the Li_*x*_CoO_2_-based neuromorphic system was demonstrated through performance evaluation in artificial neural networks. CNNs were employed to assess the reliability of analog computing utilizing measured multistate synaptic weights of Li_*x*_CoO_2_ artificial synapses. The low programming errors of the synaptic devices reported impressive results of excellent computing performance. Besides, the deep neural network-assisted crossbar array learning simulation was introduced to estimate the learning ability of Li_*x*_CoO_2_-based hardware neural networks. In the image recognition on the MNIST and Fashion MNIST data sets, the Li_*x*_CoO_2_-based artificial synapses achieved superior accuracy, surpassing to three-terminal synaptic transistors owing to fine nonlinearity and low noise. We believe that this study will not only contribute to the development of artificial two-terminal ionic synapses with the potential for structural integration and reliable performance but also pave the way toward hardware implementation of neuromorphic systems.

### Supplementary Information

Below is the link to the electronic supplementary material.

Supplementary file1 (PDF 1650 KB)
