# Supplementary material for: Two-Terminal Lithium-Mediated Artificial Synapses with Enhanced Weight Modulation for Feasible Hardware Neural Networks
Source: Nanomicro Lett. 2023 Mar 21;15:69. doi: 10.1007/s40820-023-01035-3 (PMC10030746; doi:10.1007/s40820-023-01035-3)
Supplement: Supplementary file 1 — Supplementary file1 (PDF 1650 KB) [file 40820_2023_1035_MOESM1_ESM.pdf]

Supporting Information for

Two-Terminal Lithium-Mediated Artificial Synapses with Enhanced Weight Modulation for Feasible Hardware Neural Networks

Ji Hyun Baek[1], Kyung Ju Kwak[1], Seung Ju Kim[1], Jaehyun Kim[1], Jae Young Kim[1], In hyuk Im[1], Sunyoung Lee[1], Kisuk Kang[1,]*, and Ho Won Jang[1, 2,]*

[1]Department of Materials Science and Engineering, Research Institute of Advanced Materials, Seoul National University, Seoul 08826, Republic of Korea

[2]Advanced Institute of Convergence Technology, Seoul National University, Suwon 16229, Korea

*Corresponding authors. E-mail: matlgen1@snu.ac.kr (K. Kang); hwjang@snu.ac.kr (H. W. Jang)

Supplementary Figures

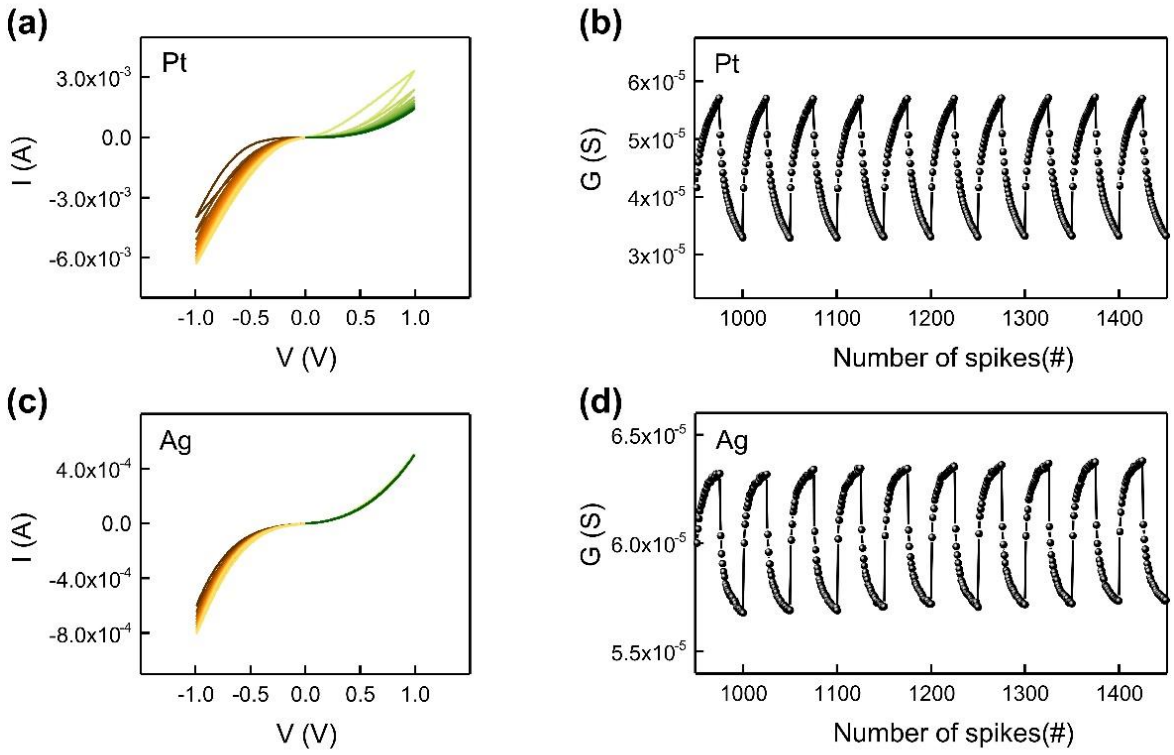

Fig. S1 Synaptic characteristics of Li_xCoO_2-based artificial synapses with different top electrodes. Analog and Spike-induced synaptic potentiation and depression of **a**, **b** Pt/Li_xCoO_2/Pt synaptic devices and **c**, **d** Ag/Li_xCoO_2/Pt synaptic devices

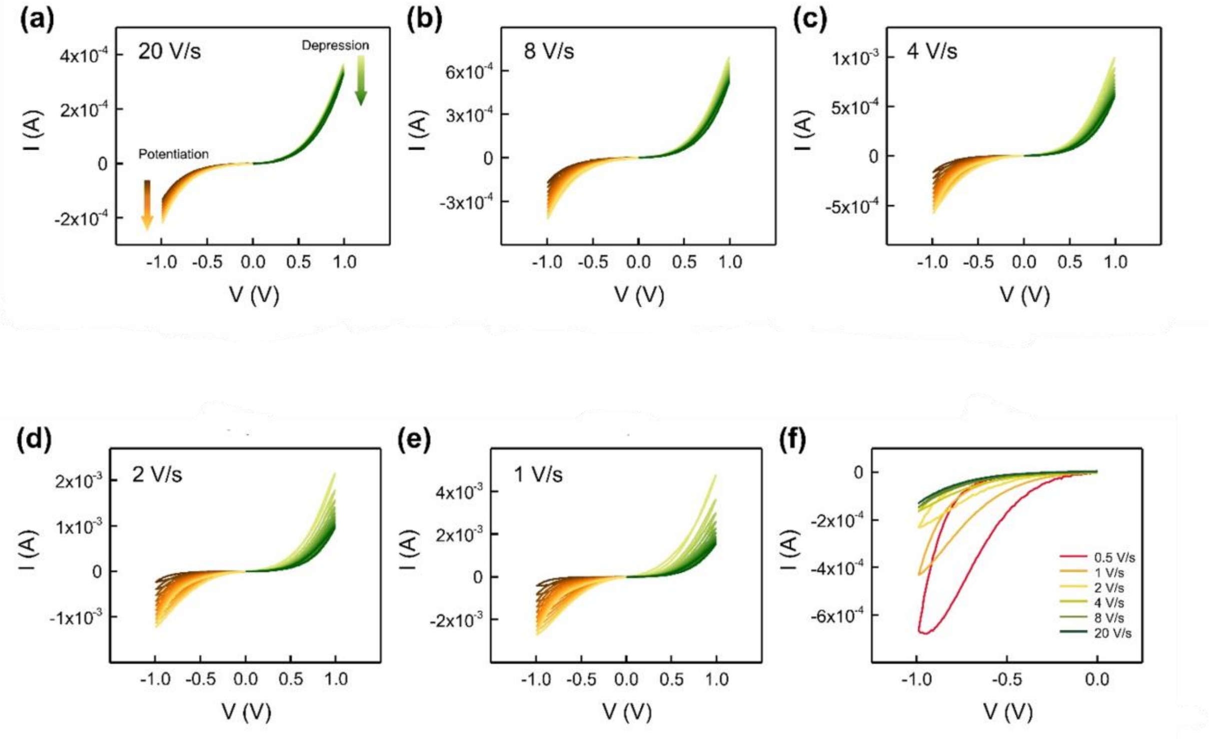

Fig. S2 Sweep rate dependence of analog synaptic behavior. **a** to **e** Analog synaptic potentiation and depression of the Au/Li_xCoO_2/Pt artificial synapses depending on different sweep rates (20 V/s to 1 V/s). **f** 1st sweep of negative sweep (0 V → -1 V → 0 V) for each sweep rate

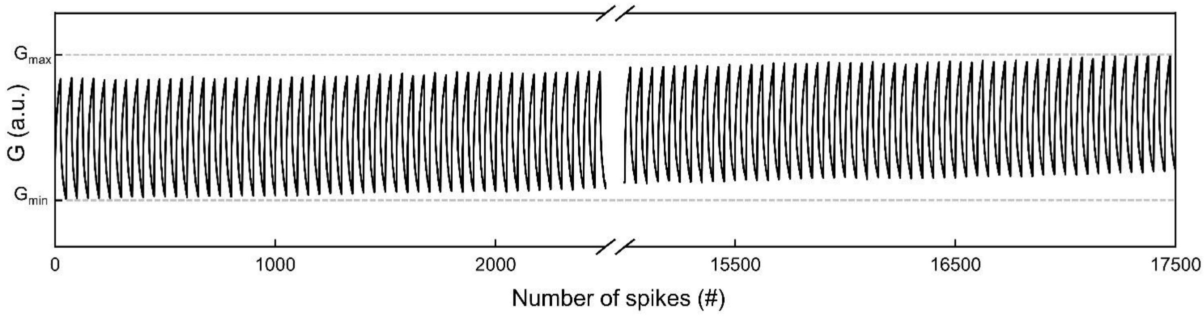

Fig. S3 Endurance of the Li_xCoO_2 artificial synapses to synaptic weight modulation. Spike-induced potentiation and depression for 350 cycles with sequential input of 25 negative and positive spikes each per cycle sequential input

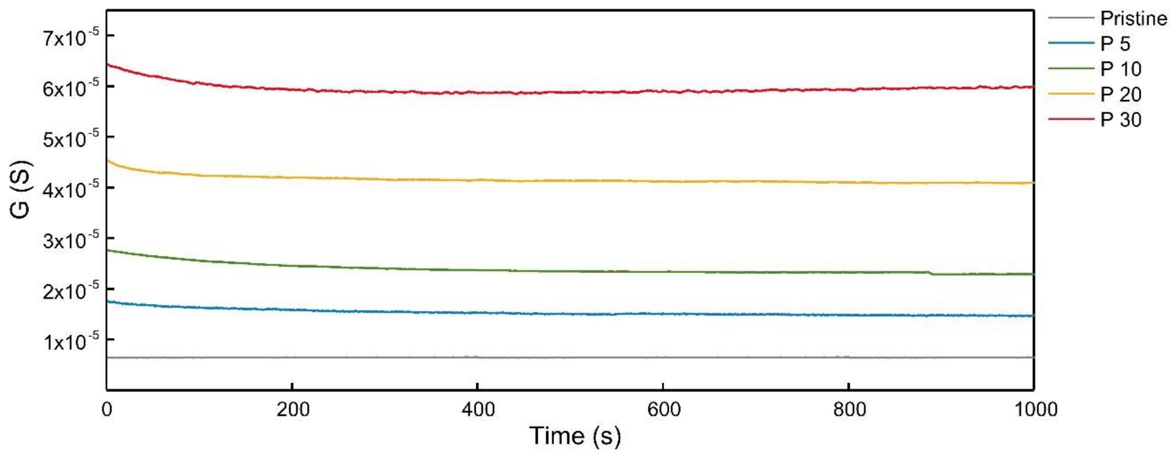

Fig. S4 Multistate LTP retention characteristics. The retention of weight updates at four distinct programming states of pristine, 10, 20, and 30 potentiations was measured for 1000 seconds

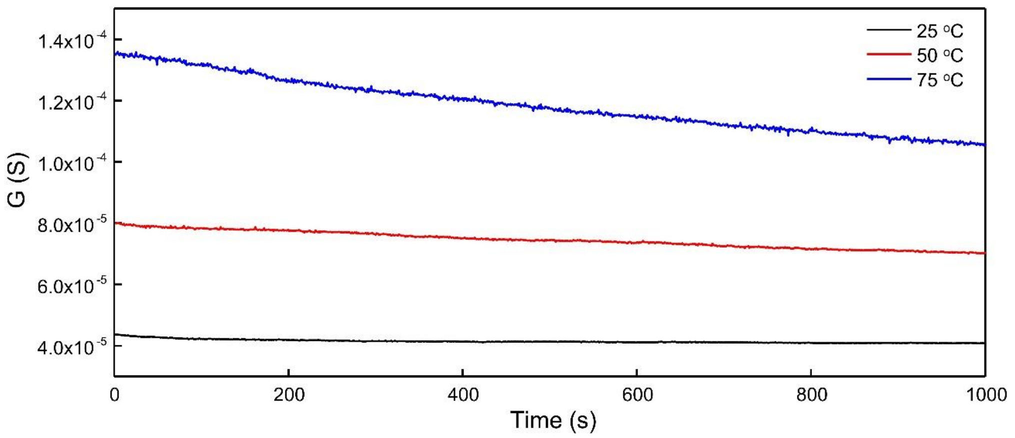

Fig. S5 Temperature-dependent retention characteristics. For 20 potentiations, LTP retention was assed at different temperatures of 25 °C, 50 °C, and 75 °C. As the temperature rises, the semiconductor Li_xCoO_2 film becomes more conductive, and the diffusion of Li ions intensifies, causing the programming state to deteriorate more

S1 Nonlinearity Estimation of LTP/LTD Curves

In this paper, two methods were applied to estimate the nonlinearity of the LTP/LTD curves. **Fig. S6** displays weight update curves of various curvatures depending on nonlinearity.

S1.1 Nonlinearity (β)

The nonlinearity (β) in the synaptic potentiation and depression can be derived from the weight update relations below, which are widely used in most synaptic devices [S1–S7].

$$G_{k+1} = G_k + \Delta G_P = G_k + \alpha_P e^{-\beta_P \frac{G_k - G_{min}}{G_{max} - G_{min}}} \quad \text{(S1)}$$

$$G_{k+1} = G_k + \Delta G_D = G_k - \alpha_D e^{-\beta_D \frac{G_{max} - G_k}{G_{max} - G_{min}}} \quad \text{(S2)}$$

Here, subscripts P and D denote weight updates in potentiation and depression. G_k and G_{k+1} indicate the conductance of the artificial synaptic device after the kth and $(k+1)^{th}$ weight control

spikes are applied, respectively. G_{min} and G_{max} are the minimum and maximum device conductance. The internal variable α represents the step size during weight update and β is the nonlinearity. The ideal β is 0, and the smaller the value, the more linear the weight update.

S1.2 Nonlinearity (α)

Some ion-based artificial synaptic devices employed nonlinearity (α) to predict the curvature of LTP/LTD curves [S8–S11].

$$G = \begin{cases} \left((G_{max}^{\alpha} - G_{min}^{\alpha}) \times \omega + G_{min}^{\alpha}\right)^{1/\alpha} & \alpha \neq 0 \\ G_{min} \times (G_{max}/G_{min})^{\omega} & \alpha = 0 \end{cases} \quad \text{(S3)}$$

Here, G_{min} and G_{max} are the minimum and maximum device conductance, respectively. The parameter α is a nonlinearity factor that determines the potentiation (α_P) or depression (α_D) behaviors. ω is an internal variable between 0 and 1 that increases (decreases) with the application of weight enhancing (weakening) spikes. For an ideal synaptic device with $\alpha_P = \alpha_D$ =1, a perfectly linear and symmetric weight update occurs. The shapes of LTP/LTD curves are convex-up if α > 1, and concave-down if α < 1.

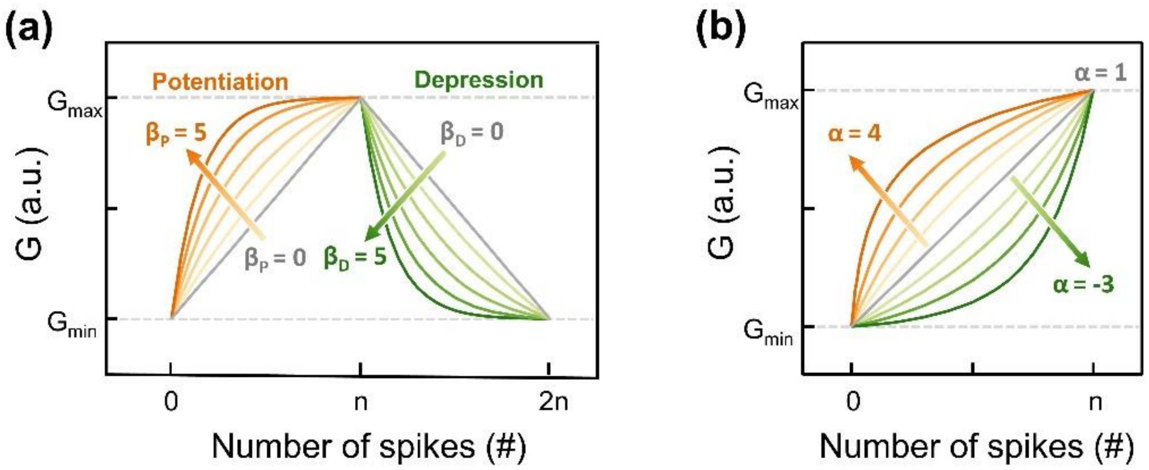

Fig. S6 LTP/LTD curves of various curvatures depending on nonlinearity. **a** The LTP/LTD curves for nonlinearity β from 0 to 5. **b** The weight update curves for nonlinearity α from -3 to 4. The shapes of LTP/LTD curves are convex-up if α > 1, and concave-down if α < 1

S2 Symmetricity Determination in Weight Updates

Figure S7 depicts the LTP/LTD curve programmed 2n times and device conductance states at the kth (orange dot) and $(2n-k)^{th}$ (green dot) weight updates. Symmetricity is defined as the reciprocal value of the symmetric error (symmetricity = symmetric error^{-1}) [S3]. The symmetric error is expressed as follows:

$$\text{Symmetric error} = \sum_{k=1}^{k=n} \frac{\left(G_N(k) - G_N(2n-k)\right)^2}{n} = \sum_{k=1}^{k=n} \frac{\left((G(k)-G_{min}) - (G(2n-k)-G_{min})\right)^2}{n(G_{max}-G_{min})^2}$$
$$= \sum_{k=1}^{k=n} \frac{\left(G(k)-G(2n-k)\right)^2}{n(G_{max}-G_{min})^2}, \; where \; G_N(k) = \frac{G(k)-G_{min}}{G_{max}-G_{min}} \quad \text{(S4)}$$

Here, G_N, G_{max}, and G_{min} signify the normalized, the maximum, and the minimum value of the device conductance, respectively. The complete asymmetry (symmetry error = ∞) and perfect symmetry (symmetry error = 0) cases of the LTP/LTD curves are illustrated in **Fig. 7**.

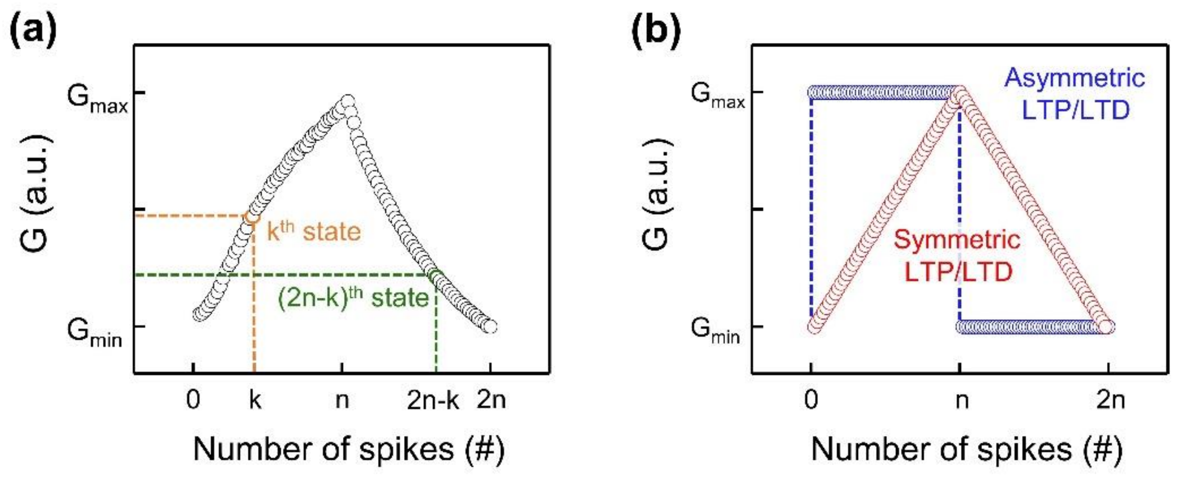

Fig. S7 Symmetricity determination in weight updates. **a** LTP/LTD curve programmed 2n times and device conductance states at the kth (orange dot) and $(2n-k)^{th}$ (green dot) weight updates. **b** The complete asymmetry (symmetry error = ∞) and perfect symmetry (symmetry error = 0) cases of the LTP/LTD curves

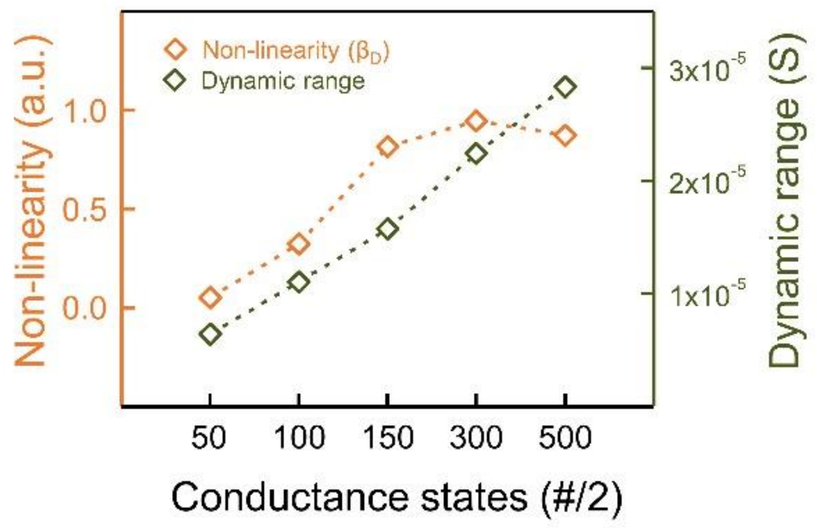

Fig. S8 The nonlinearity and dynamic range of depression step of SNDP in **Fig. 3a**. The nonlinearities (β_D) and dynamic ranges extracted from LTD curves with respect to the different number of weight control spikes

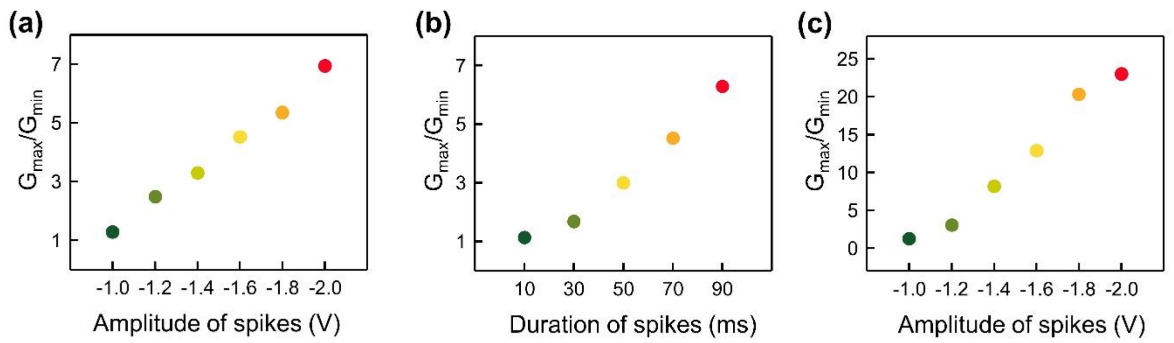

Fig. S9 Ratios of synaptic efficacy in different spiking regimes. The ratio of the minimum conductance measured in the initial state to the maximum conductance obtained after potentiation in **a** SVDP, **b** SWDP, and **c** SVWDP in **Fig. 4**

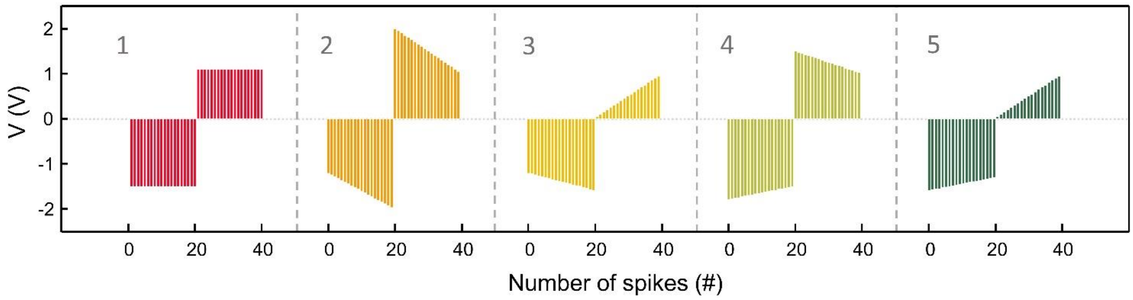

Fig. S10 The graphical illustration of spike trains used for LTP/LTD customization in **Fig. 4e**

S3 Asymmetric Ratio Analysis of LTP/LTD Curves

The asymmetric ratio (AR) between the two successive LTP and LTD curves, each with n weight updates, is defined as

$$AR = \left[\frac{max|G_P(k)| - max|G_D(k)|}{G_{P(n)} - G_{D(n)}}\right] \; for\ k = 1\ to\ n \tag{S5}$$

where $|G_P(k)|$ and $|G_D(k)|$ indicate the average conductance values during potentiation and depression, respectively [S8,S9,S12]. The $G_P(n)$ and $G_D(n)$ represent the device conductance after programmed n times. The asymmetric ratio should be zero for an ideal case.

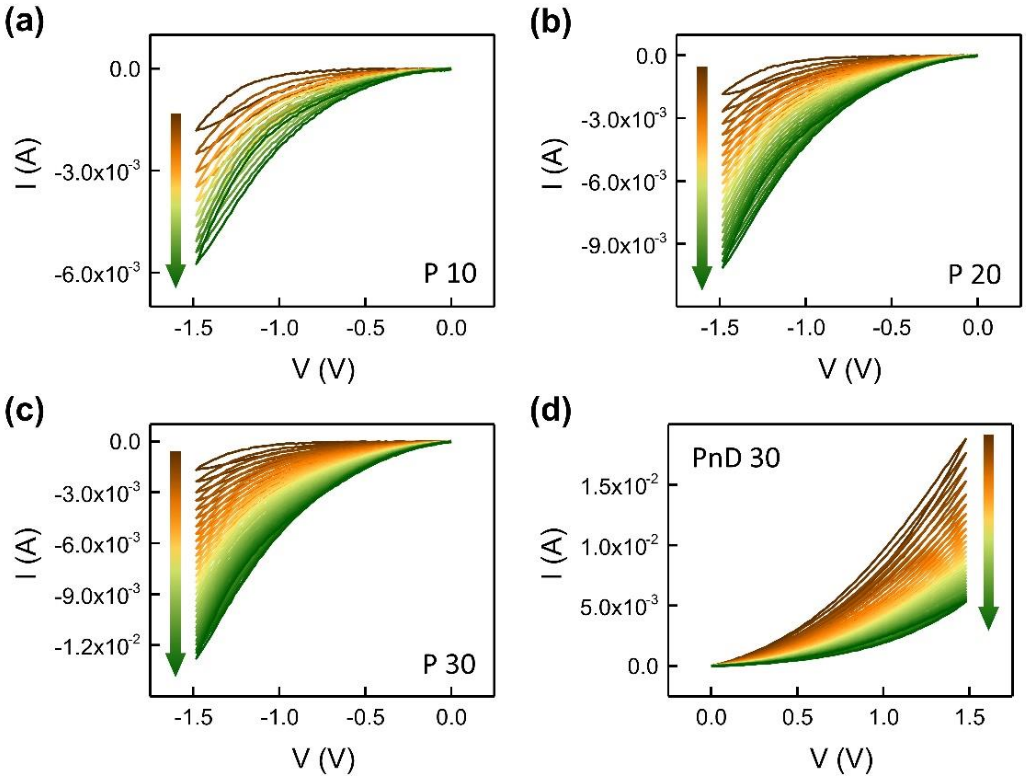

Fig. S11 Four synaptic activities via analog voltage sweeps. Potentiation of the Au/Li_xCoO_2/Pt synaptic device during **a** 10, **b** 20, and **c** 30 consecutive negative voltage sweeps (0 V → -1.5 V → 0 V), respectively, and **d** depression after 30 times of potentiation along 30 positive voltage sweeps (0 V → 1.5 V → 0 V)

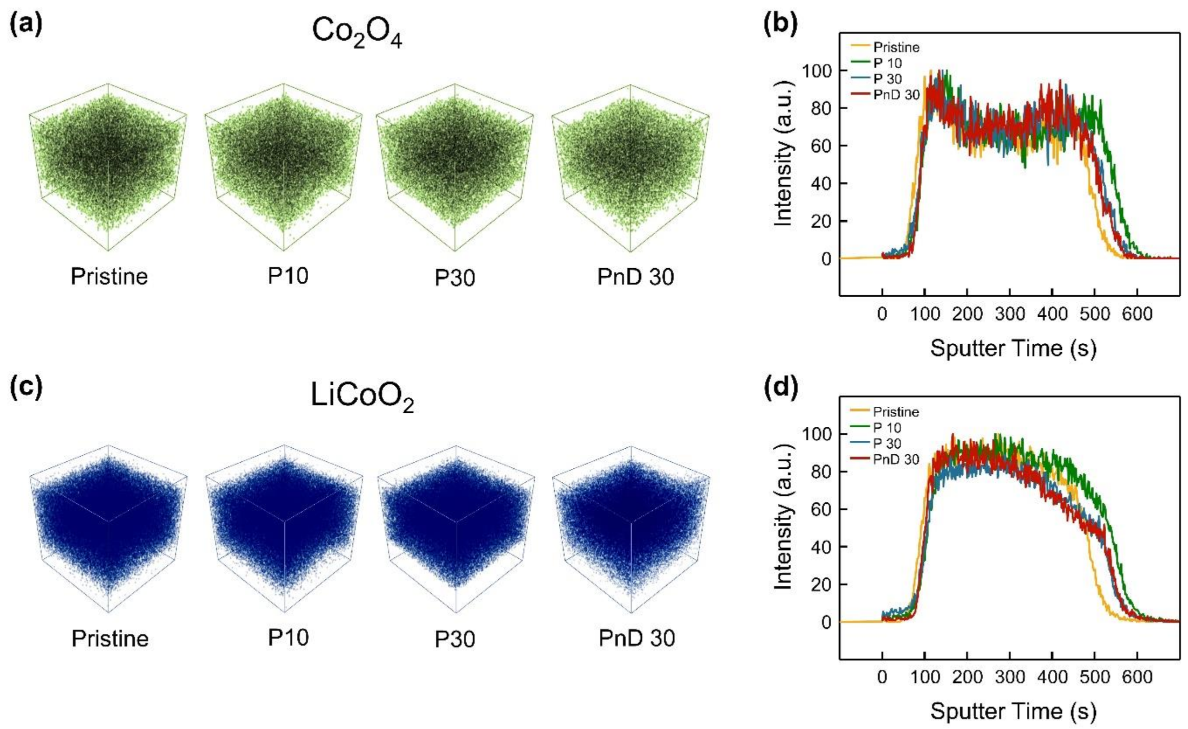

Fig. S12 ToF-SIMS analysis of the Li_xCoO_2 framework at different synaptic weight states. ToF-SIMS 3D mappings and depth profiles of **a**, **b** the Co_2O_4 and the **c**, **d** $LiCoO_2$ elements in Au/Li_xCoO_2/Pt structure at four different weight states. Unlike Li cations, both elements do not penetrate into the Au top electrode during weight control but maintain the framework $LiCoO_2$ crystal structure

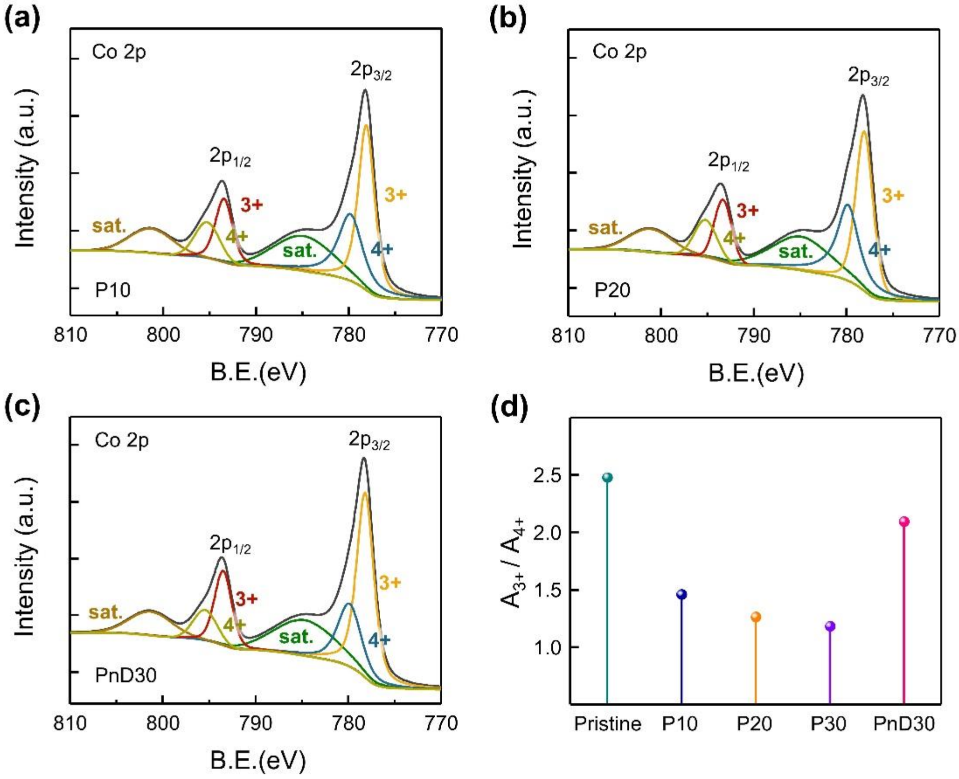

Fig. S13 XPS analysis of Co ion at different synaptic weight states. XPS spectra of the Co 2p region for Li_xCoO_2 film after **a** 10 potentiations, **b** 20 potentiations, and **c** 30 depressions

following 30 potentiations. **d** The area ratios of Co^{4+}/Co^{3+} for five different synaptic weight states

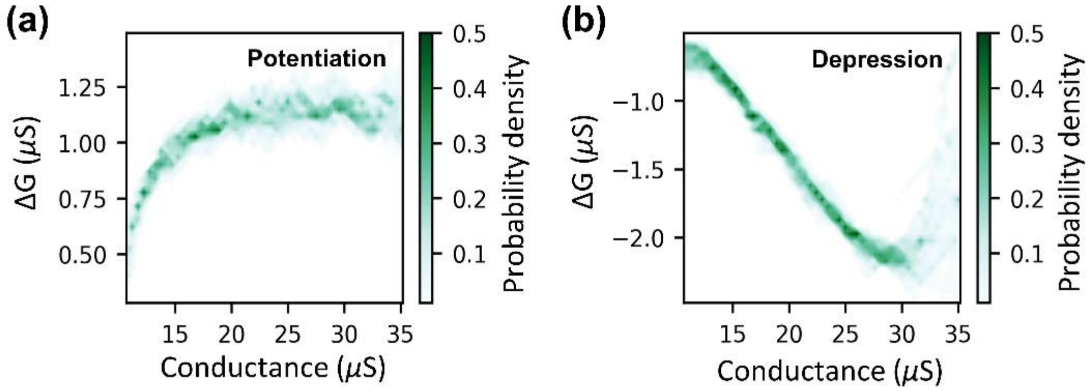

Fig. S14 Statistics of weight updates in Li_xCoO_2 artificial synapses. Heat map of the ΔG vs. G during **a** potentiation and **b** depression

S4 CNN-Based Image Recognition Simulation

A convolutional neural network (CNN) is a class of deep learning algorithms specialized in processing pixel data for image recognition [S13]. **Fig. S15a** represents a schematic of image inference for ImageNet in ResNet50-v1.5, a 50-layer deep CNN model, carrying feature extraction and inference [S14, S15]. In the feature extraction step, hierarchical patterns with complexity are scaled down and assembled into simpler and smaller patterns embossed on the filter. The cumulated data processed stepwise is flattened in one dimension in the inference stage and fed into a fully connected layer. The input image is classified by returning the most probable result among 1000 classes of ImageNet via the activation function for the input weights. **Fig. S15b** depicts the diagram of data processing and algorithm architecture of the ResNet50-v1.5 model. The ResNet50-v1.5 model consists of one max pooling layer, one average pooling layer, and five convolutional stages comprised of several residual blocks which are the foundational building blocks of the ResNet architecture [S16]. The first residual block of each stage is a convolutional block that halves the input scale. **Fig. S15c** presents the topology of a residual block, also known as an identity block, with a skip connection [S17]. The identity function is used as a shortcut to permit gradients to propagate directly into a deeper layer in networks, bypassing non-linear activation functions. When the desired underlying mapping is defined as $H(x_l)$, the goal is to optimize the final equation $H(x_l)$, which is formulated as $H(x_l) = F(x_l) + x_l$. Here, $F(x_l)$ denotes the form of the input x_l after going through the convolutional layers, batch normalizations, and activation functions ReLU. At this time, since the x_l is referenced as an input value, the optimization is only discussed on $F(x_l) = H(x_l) - x_l$. The $F(x_l)$ behaves like a residual, thus the name 'residual block.'

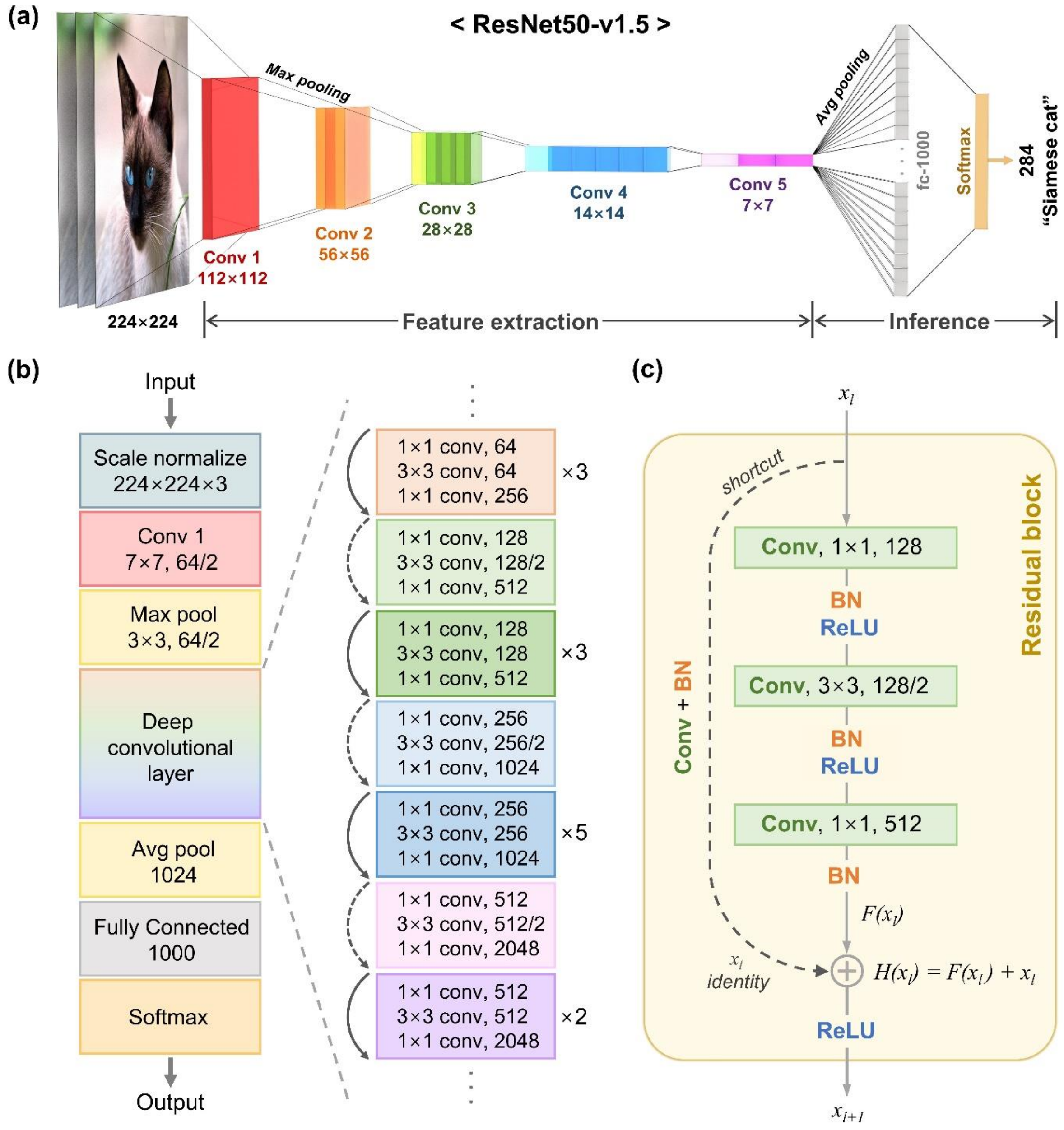

Fig. S15 Image recognition of Li_xCoO_2-based neuromorphic system employing CNNs. **a** Schematic diagram of image recognition in ResNet50-v1.5 carrying feature extraction and inference. **b** The data processing flow and architecture of residual network with 50 layers deep. **c** A building block of residual learning with identity mapping

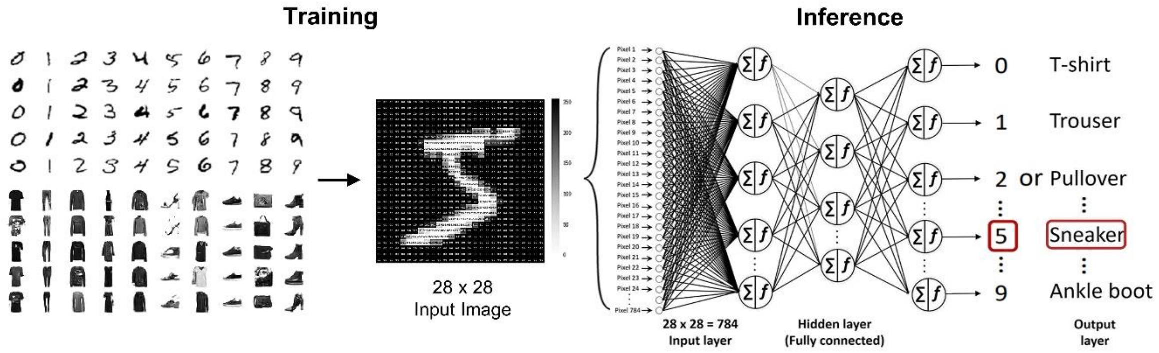

Fig. S16 Schematic diagram of data processing flow in deep neural networks for image recognition

S5 MLPs-Based Image Recognition Simulation

The file type [S18], MNIST [S19], and fashion MNIST [S20] datasets were chosen for image recognition. The file type database is classified into 9 categories (AES-256, GZIP, ELF, DOC, PDF, GIF, JPG, PNG, and HTMP). Each data type can be identified by evaluating its performance across three input spaces: the byte probability distribution, the power spectral density, and the sliding window entropy of a sequence of bytes in the file [S18]. The large MNIST and fashion MNIST data sets consist of ten types of handwritten digits (0−9) and ten types of Zalando's article images (T-shirts, trousers, pullovers, dresses, coats, sandals, shirts, sneakers, bags, and ankle boots), respectively. In the image recognition to large MNIST and fashion MNIST, the 28 × 28 pixels of images correspond to the 28 × 28 pre-neurons and serve as an input layer in the multilayer perceptron. The monochrome images of MNIST and fashion MNIST were transformed to grayscale values ranging from 0 to 255 and supplied to the pre-neurons input. There is a single hidden layer with 300 hidden neurons between the input and output layers. The output layer has 10 output neurons, each corresponding to one of 10 types of images in the input data set. All neurons in each layer are fully connected to all neurons in the following layer via synapses. Each neural network was trained for 40 epochs, with each epoch exploring an optimal inferred model by training and testing on assigned training sets at random. The training was individually done on an allocated number of training sets of 100, 1,000, and 10,000 images.

Forward propagation proceeds through the activation of neurons that transmits a signal from the previous neuron to the next according to the synaptic weight. As a nonlinear activation function, the sigmoid function controls the firing of neurons. The learning algorithm described above is programmed in Python.

Table S1 Synaptic weight modulation of diverse artificial synaptic devices

Device structure	Mechanism	Weight control spikes		Linearity	Refs.
		Potentiation	Depression		
ITO/MXene-ZnO /Al	Conductive filament	153 W/cm^2 150 ms	−8 V 100 ms	Good	[S21]
Au/$CsCu_2I_3$/ITO	Conductive filament	−2 V 5 ms	2 V 5 ms	Good	[S2]
G/DEME-TFSI/VO_2/S-D	Phase transition	84 mW/cm^2 10 s	−2.5 V 10 s	Good	[S5]
Au/Li_xMoS_2/Au	Phase transition	4 V 1 ms	−4 V 1 ms	Poor	[S22]
Ag/WO_{3-x}/WSe2/Gr	Vacancy migration	−4 V 40 ms	0.5 V 5 ms	Poor	[S23]
Al/ IGZO/ Al_2O_3/TiO_2/SiO_2/P^+-Si	Vacancy migration	+3 V 100 ms	−3 V 100 ms	Moderate	[S24]
Au/$PEA_2MA_{n-1}Pb_nI_{3n+1}$/ITO	Ion migration	+1 V 1 ms	−1V 1 ms	Moderate	[S1]
Ion-gel/Gr/WS2/Au	Ion migration	+0.5 V 50 ms	−-0.5V 50 ms	Moderate	[S7]
Al/GPSi/IGZO/ITO	Polarization change	+2 V 200 ms	−2 V 200 ms	Moderate	[S25]
Au/PVDF-TrFE/Au	Polarization change	+30V 500 ms	−30 V 500 ms	Moderate	[S26]
Au/WSe_2/*h*-BN/Au	Charge trapping	+0.3 V 20 ms	−0.3 V 20 ms	Moderate	[S4]
Au/WSe_2/*h*-BN// MoS_2/*h*-BN/Au	Charge trapping	+1 V 20 ms	+ 1 V 20 ms	Moderate	[S3]
Si/LiPON/Li_xCoO_2/Pt	Li^+ intercalation	−75 mV 2 s	+75 mV 2 s	Good	[S27]

Pt/Si/$Li_3PO_xSe_x/LiCoO_2$/Pt	Li^+ intercalation	+1.5 V<br>1 s	−1.5 V<br>1 s	Good	[S8]
Au/Li_xCoO_2/Pt	Li^+ intercalation	+1.5 V<br>10 ms	-1.1 V<br>10 ms	Good	**This work**

Table S2 The spike conditions of LTP/LTD customization in **Fig. 4 e**

Shape number	Potentiation V (V)	Depression V (V)	Pulse duration (ms)
1	-1.5	1.1	10
2	-1.2 → -2.0	2.0 → 1.0	10
3	-1.2 → -1.6	0.0 → 1.0	10
4	-1.8 → -1.5	-1.5 → 1.0	10
5	-1.6 → -1.3	0.0 → 1.0	10

Table S3 Details of the datasets and neural network models used for image recognition

Datasets	Input shape	# of classes	Neural networks model		Note	Refs.
File types	(16, 16, 1)	9	DNN	(256, 150, 9)	-	[S18]
Large MNIST	(28, 28, 1)	10		(784, 300, 10)	-	[S19]
Fashion MNIST	(28, 28, 1)	10		(784, 300, 10)	-	[S20]
Large MNIST	(28, 28, 1)	10	CNN	CNN6 v2	Four convolutional + two dense layers, 119.3K weights.	[S19]
CIFAR-10	(32, 32, 3)	10		ResNet56	Follows the architecture in Ref. [S17]. 861.8K weights.	[S28]
CIFAR-100	(32, 32, 3)	100		ResNet56	Follows the architecture in Ref. [S17] with 4× more channels (16×weights).	[S29]
ImageNet	(224, 224, 3)	1000		ResNet50-v1.5	Follows the architecture in Ref. [S16].	[S15]

Supplementary References

[S1] S.J. Kim, T.H. Lee, J.M. Yang, J.W. Yang, Y.J. Lee et al., Vertically aligned two-dimensional halide perovskites for reliably operable artificial synapses. Mater. Today **52**, 19–30 (2022). https://doi.org/10.1016/j.mattod.2021.10.035

[S2] K.J. Kwak, J.H. Baek, D.E. Lee, I. Im, J. Kim et al., Ambient stable all inorganic $CsCu_2I_3$ artificial synapses for neurocomputing. Nano Lett. **22**(14), 6010-6017 (2022). https://doi.org/10.1021/acs.nanolett.2c01272

[S3] S. Seo, B.S. Kang, J.J. Lee, H.J. Ryu, S. Kim et al., Artificial van der Waals hybrid synapse and its application to acoustic pattern recognition. Nat. Commun. **11**, 3936 (2020). https://doi.org/10.1038/s41467-020-17849-3

[S4] S. Seo, S.H. Jo, S. Kim, J. Shim, S. Oh et al., Artificial optic-neural synapse for colored and color-mixed pattern recognition. Nat. Commun. **9**, 5106 (2018). https://doi.org/10.1038/s41467-018-07572-5

[S5] G. Li, D. Xie, H. Zhong, Z. Zhang, X. Fu et al., Photo-induced non-volatile VO_2 phase transition for neuromorphic ultraviolet sensors. Nat. Commun. **13**, 1729 (2022). https://doi.org/10.1038/s41467-022-29456-5

[S6] S. Park, S. Oh, D. Lee, J.H. Park, Ferro-floating memory: dual-mode ferroelectric floating memory and its application to in-memory computing. InfoMat **4**, e12367 (2022). https://doi.org/10.1002/inf2.12367

[S7] S. Oh, J.H. Lee, S. Seo, H. Choo, D. Lee et al., Electrolyte-gated vertical synapse array based on van der Waals heterostructure for parallel computing. Adv. Sci. **9**, 2103808 (2022). https://doi.org/10.1002/advs.202103808

[S8] R.D. Nikam, M. Kwak, J. Lee, K.G. Rajput, W. Banerjee et al., Near ideal synaptic functionalities in Li ion synaptic transistor using $Li_3PO_xSe_x$ electrolyte with high ionic conductivity. Sci. Rep. **9**, 18883 (2019). https://doi.org/10.1038/s41598-019-55310-8

[S9] R.D. Nikam, M. Kwak, J. Lee, K.G. Rajput, H. Hwang, Controlled ionic tunneling in lithium nanoionic synaptic transistor through atomically thin graphene layer for neuromorphic computing. Adv. Electron. Mater. **6**(2), 1901100 (2020). https://doi.org/10.1002/aelm.201901100

[S10] J.W. Jang, S. Park, Y.H. Jeong, H. Hwang, ReRAM-based synaptic device for neuromorphic computing. Proc. IEEE Int. Symp. Circuits Syst., Melbourne, VIC, Australia, 1054–1057 (June, 2014). https://doi.org/10.1109/ISCAS.2014.6865320

[S11] J. Jang, S. Park, G.W. Burr, H. Hwang, Y. Jeong, Optimization of conductance change in $Pr_{1-x}Ca_xMnO_3$-based synaptic devices for neuromorphic systems. IEEE Electron Device Lett. **36**, 457–459 (2015). https://doi.org/10.1109/LED.2015.2418342

[S12] C.S. Yang, D.S. Shang, N. Liu, E.J. Fuller, S. Agrawal et al., All-solid-state synaptic transistor with ultralow conductance for neuromorphic computing. Adv. Funct. Mater. **28**(42), 1804170 (2018). https://doi.org/10.1002/adfm.201804170

[S13] R. Yamashita, M. Nishio, R.K.G. Do, K. Togashi, Convolutional neural networks: an overview and application in radiology. Insights Imaging **9**, 611–629 (2018). https://doi.org/10.1007/s13244-018-0639-9

[S14] A. Krizhevsky, I. Sutskever, G.E. Hinton, Imagenet classification with deep convolutional neural networks. Commun. ACM **60**, 84–90 (2017).

[S15] J. Deng, W. Dong, R. Socher, L.J. Li, K. Li et al., ImageNet: a large-scale hierarchical image database. 2009 IEEE Conf. Comput. Vis. Pattern Recognit., Miami, FL, USA, 248–255 (June, 2009). https://doi.org/10.1109/CVPR.2009.5206848

[S16] V.J. Reddi, C. Cheng, D. Kanter, P. Mattson, G. Schmuelling et al., MLPerf inference benchmark. Proc. Int. Symp. Comput. Archit., Valencia, Spain, 446–459 (2020). https://doi.org/10.1109/ISCA45697.2020.00045

[S17] K. He, X. Zhang, S. Ren, J. Sun, Deep residual learning for image recognition. Proc. IEEE Comput. Soc. Conf. Comput. Vis. Pattern Recognit., Las Vegas, NV, USA, 770–778 (2016). https://doi.org/10.1109/CVPR.2016.90

[S18] J.A. Cox, C.D. James, J.B. Aimone, A signal processing approach for cyber data classification with deep neural networks. Procedia Comput. Sci. **61**, 349–354 (2015). https://doi.org/10.1016/j.procs.2015.09.156

[S19] Y. LeCun, C. Cortes, The MNISTdatabase of handwritten digit images for machine learning research. (2010). http://yann.lecun.com/exdb/mnist

[S20] H. Xiao, K. Rasul, R. Vollgraf, Fashion-MNIST: a novel image dataset for benchmarking machine learning algorithms. arXiv, 1708.07747 (2017). https://doi.org/10.48550/arXiv.1708.07747

[S21] Y. Wang, Y. Gong, L. Yang, Z. Xiong, Z. Lv et al., MXene-ZnO memristor for multimodal in-sensor computing. Adv. Funct. Mater. **31**(21), 2100144 (2021). https://doi.org/10.1002/adfm.202100144

[S22] X. Zhu, D. Li, X. Liang, W.D. Lu, Ionic modulation and ionic coupling effects in MoS_2 devices for neuromorphic computing. Nat. Mater. **18**, 141–148 (2019). https://doi.org/10.1038/s41563-018-0248-5

[S23] W. Huh, S. Jang, J.Y. Lee, D. Lee, D. Lee et al., Synaptic barristor based on phase-engineered 2D heterostructures. Adv. Mater. **30**(35), 1801447 (2018). https://doi.org/10.1002/adma.201801447

[S24] H. Park, S. Oh, S.H. Jeong, O. Kwon, H.Y. Seo et al., Dual-terminal stimulated heterosynaptic plasticity of IGZO memtransistor with Al_2O_3/TiO_2 double-oxide structure. ACS Appl. Electron. Mater. **4**(6), 2923–2932 (2022). https://doi.org/10.1021/acsaelm.2c00393

[S25] S. Kim, S. Kim, D.H. Ho, D.G. Roe, Y.J. Choi et al., Neurorobotic approaches to emulate human motor control with the integration of artificial synapse. Sci. Adv. **8**, eabo3326 (2022). https://doi.org/10.1126/sciadv.abo3326

[S26] S. Jang, S. Jang, E.H. Lee, M. Kang, G. Wang et al., Ultrathin conformable organic artificial synapse for wearable intelligent device applications. ACS Appl. Mater. Interfaces. **11**(1), 1071–1080 (2019). https://doi.org/10.1021/acsami.8b12092

[S27] E.J. Fuller, F.E. Gabaly, F. Léonard, S. Agarwal, S.J. Plimpton et al., Li-ion synaptic transistor for low power analog computing. Adv. Mater. **29**(4), 1604310 (2017).https://doi.org/10.1002/adma.201604310

[S28] A. Krizhevsky, V. Nair, G. Hinton, CIFAR-10 (Canadian Institute for Advanced Research). http://www.cs.toronto.edu/~kriz/cifar.html

[S29] A.Krizhevsky, V. Nair, G. Hinton, CIFAR-100 (Canadian Institute for Advanced Research). http://www.cs.toronto.edu/~kriz/cifar.html
